# Integrative sequence and tissue expression profiling of chicken and mammalian aquaporins

**DOI:** 10.1186/1471-2164-10-S2-S7

**Published:** 2009-07-14

**Authors:** Raphael D Isokpehi, Rajendram V Rajnarayanan, Cynthia D Jeffries, Tolulola O Oyeleye, Hari HP Cohly

**Affiliations:** 1 Center for Bioinformatics & Computational Biology, Department of Biology, Jackson State University, PO Box 18540, Jackson MS 39217, USA; 2 Department of Chemistry, Tougaloo College, Jackson MS 39174, USA

## Abstract

**Background:**

Proteins that selectively transport water across the membranes of cells are recognized as important in the normal functioning of the body systems of vertebrates. There are 13 known mammalian aquaporins (AQP0 to AQP12), some of which have been shown to have unexpected cellular roles beyond transmembrane water transport. The availability of non-mammalian vertebrate animal models has the potential to provide insight into the emergence of diverse function in the aquaporins. The domesticated chicken (*Gallus gallus*) is the premier avian model for biological research; however, only a limited number of studies have compared chicken and mammalian aquaporins. The identification of aquaporins that share functional motifs or are expressed in the same tissues in human and chicken could allow the further functional analyses of homologous aquaporins in both species. We hypothesize that integrative analyses of protein sequences and body site expression of human, mouse, rat and chicken aquaporins has the potential to yield novel biological hypotheses about the unexpected cellular roles of aquaporins beyond transmembrane water transport.

**Results:**

A total of 76 aquaporin transcript models derived from 47 aquaporin genes were obtained for human, mouse, rat and chicken. Eleven body sites (brain, connective tissue, head, heart, liver, muscle, ovary, pancreas, small intestine, spleen and testis) were identified in which there is suggested expression of at least one mammalian and one chicken aquaporin. This study demonstrates that modern on-line analysis tools, a novel matrix integration technique, and the availability of the chicken genome for comparative genomics and expression analysis enables hypothesis generation in several important areas including: (i) alternative transcription and speciation effects on the conservation of functional motifs in vertebrate aquaporins; (ii) the emergence of basolateral targeting in mammalian species; (iii) the potential of the cysteine-rich AQP11 as a possible target in the pathophysiology of neurodegenerative disorders such as autism that involve Purkinje cells; and (iv) possible impairment of function of pancreas-expressed AQP12 during pancreatotropic necrosis in avian influenza virus infection.

**Conclusion:**

The investigation of aquaporin function in chicken and mammalian species has the potential to accelerate the discovery of novel knowledge of aquaporins in both avian and mammalian species.

## Background

**Figure 1:**
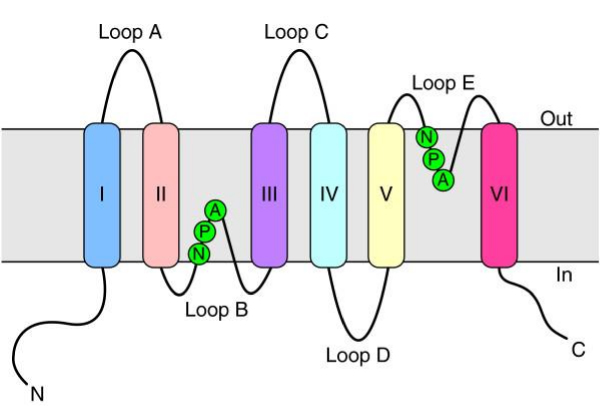
**Topology of an aquaporin protein within the membrane**. The protein consists of six transmembrane helices (I-VI) connected by five loops (A-E) and includes two internal tandem repeats (I-III and IV-VI, respectively). Loops B and E, containing the conserved NPA motifs (in the single-letter amino-acid code), form short α helices that fold back into the membrane from opposite sides. C, carboxyl terminus; N, amino terminus. Figure reproduced from [6].

Proteins that selectively transport water across the membranes of cells are recognized as important in normal functioning of the body systems of vertebrates. These homologous proteins are collectively referred to as aquaporins and include a subset called aquaglyceroporins that are able to transport glycerol, urea and other small solutes in addition to water [1]. There are 13 known mammalian aquaporins (AQP0 to AQP12). These aquaporins vary in tissue and developmental expression across mammalian species and unexpected cellular roles for the aquaporins beyond transmembrane water transport have been identified [2]. However, the cellular and molecular strategies for these roles are not completely understood. The water-only aquaporins are AQP0, AQP1, AQP2, AQP4, AQP5, AQP6 and AQP8, while aquaglyceroporins are AQP3, AQP7, AQP9 and AQP10 [1]. The transport specificities and roles of AQP11 and AQP12 in health and disease are not completely described [1,3-5]. Aquaporins typically have six transmembrane regions and five loops (A to E) with two characteristic Asparagine-Proline-Alanine (NPA) motifs in loops B and E (Figure 1) [6]. Based on the number of citations in PubMed, avian aquaporins have been poorly investigated compared to those of human, mouse and rat [7].

The domesticated chicken *Gallus gallus *is the premier avian model for biological research [8-10]. Furthermore, the publication of the chicken genome and availability of large-scale gene expression data presents new opportunities to compare the expression of avian and mammalian aquaporin genes. Mammalian aquaporins function in diverse biological processes including development of lens, inner ear, teeth and oral facial tissues; maintenance of sperm motility; synthesis and secretion of milk; and urine concentration. In addition, malfunction of mammalian aquaporins have been implicated in diverse disease processes such as altered fat metabolism, brain edema, cancer, congenital cataract, dry mouth, dry skin, infertility, hearing loss, nephrogenic diabetes, renal failure, and wet lung syndrome [11]. This broad range of pathologies linked to altered aquaporin expression has further supported the potential of aquaporins as drug targets for water-transport related disorders [12].

Apart from transmembrane/epithelial water transport, recently suggested unexpected cellular roles of mammalian aquaporins include cell migration, cell volume regulation, mitochondria metabolism, neural signal transduction, renal glycerol clearance and vesicular swelling [11,13]. The co-expression of several members of the aquaporin gene family in the same tissue such as small intestine [14] makes it difficult to determine their individual role apart from trans-epithelial water transport. It is not clear whether alternative transcription/splicing of an aquaporin gene to produce more than one isoform contributes to these unexpected cellular roles. Mice with deficient or mutated aquaporin are currently used as models to study novel cellular roles of human aquaporins [11]. There is an increasing need to have alternative non-mammalian models for aquaporin function in humans [14]. The chick optic tectum has been used to investigate the role of AQP4 in blood brain barrier development [15]. Furthermore, in chick lens development, AQP0 interacts with lens-fiber gap junctions during lens development [16].

There are a limited numbers of reports that have compared the body site expression of chicken and mammalian aquaporins. Comparison of nucleotide sequences of chicken AQP2, AQP4 and AQP5 to their rat and human orthologs has revealed an overall identity of 75–90% and similarity in tissue distribution [7]. AQP9 has not been shown to be expressed in mammalian kidney, but recently found expressed in young chicken kidney [17]. In addition, water-deprivation in early development of rodents and chicken results in upregulation of the kidney AQP2 [17,18]. The distribution of AQP4 in the circumventricular organs of chicken and rat brains have been compared [19]. Taken together, the identification of aquaporins that share sequence similarity or are expressed in identical tissues in human and chicken could allow the further functional analyses of aquaporins in avian species.

The objectives of the investigation reported in this article were to (1) determine the impact of alternative transcription and speciation on functional motifs of aquaporin gene transcript models predicted from the genomes of human, mouse, rat and chicken; and (2) determine body sites common to human, mouse, rat and chicken with suggested aquaporin expression. We hypothesize that integrative analyses of protein sequences and body site expression of human, mouse, rat and chicken aquaporins has the potential to yield novel biological hypotheses about the unexpected cellular roles of aquaporins beyond transmembrane water transport. Our high-throughput comparative sequence analysis revealed the potential impact of alternative transcription and speciation on the conservation of functional motifs in human, mouse, rat and chicken aquaporins. Furthermore, by using controlled vocabulary of terms describing body sites in the UniGene database, an integrated view of suggested tissue expression of aquaporins for these four organisms was generated.

The integrative analyses of protein sequences and tissue expression profiles presented in this article provides novel insights into the potential function of cysteine-rich AQP11 in the pathophysiology of autism and evidence for involvement of pancreas-expressed AQP12 in the pathology of highly pathogenic avian influenza virus infections. The investigation of aquaporin function in chicken could accelerate the discovery of novel knowledge on human aquaporins especially during early development.

## Results

### Comparison of predicted aquaporin transcripts from human, mouse, rat and chicken

In order to determine the impact of alternative transcription as well as speciation on the conservation of functional motifs in aquaporins, we first compiled the aquaporin types with entries in the Entrez Gene [20] and Ensembl [21] genomic resources (Table 1) for human, mouse, rat and chicken. This dataset provided the basis for comparative sequence analysis of the transcripts predicted in the Ensembl genome resource. Multiple sequence alignment was performed on each aquaporin type in which at least one mammalian and one chicken transcript were available. A total of 76 sequences were retrieved from the Ensembl. Multiple sequence alignments for each aquaporin type are available as Additional File 1. A summary of the number of transcripts examined for 9 aquaporin types and key observations from sequence alignment are presented in Table 2. The impact of alternative transcription and speciation of these vertebrate aquaporins on the conservation of the two water-transport motifs is summarized in Table 3. We further describe below the results obtained for AQP3, AQP4, AQP11 and AQP12.

**Table 1:** Aquaporin entries in Entrez Gene, UniGene, and Ensembl genomic resources

AQP	Human			Mouse			Rat			Chicken		
	Entrez Gene	Unigene	Ensembl*	Entrez Gene	Unigene	Ensembl*	Entrez Gene	Unigene	Ensembl*	Entrez Gene	Unigene	Ensembl*
**AQP 0**	4284	Hs.574026	135517	17339	Mm.31625	25389	25480	Rn.23532	3132	374124	Gga.67	-
**AQP 1**	358	Hs.76152	106125	11826	Mm.18625	4655	25240	Rn.1618	11648	420384	Gga.2680	5209
**AQP 2**	359	Hs.130730	167580	11827	Mm.20206	23013	25386	Rn.90076	297	-	-	-
**AQP 3**	360	Hs.234642	165272	11828	Mm.34043	28435	65133	Rn.11109	9797	426894	Gga.23800	2452
												
**AQP 4**	361	Hs.315369	171885	11829	Mm.250786	24411	25293	Rn.90091	16043	421088	Gga.11374	15128
**AQP 5**	362	Hs.298023	161798	11830	Mm.45580	44217	25241	Rn.10066	17685	431305	Gga.6412	2720
												
**AQP 6**	363	Hs.54505	86159	11831	Mm.202309	43144	29170	Rn.48667	296	-	-	10260
**AQP 7**	364	Hs.455323	165269	11832	Mm.8728	28427	29171	Rn.11111	9686	426892	Gga.21944	18534
												
**AQP 8**	343	Hs.176658	103375	11833	Mm.273175	30762	29172	Rn.6315	14652	416566	Gga.6178	5988
												
**AQP 9**	366	Hs.104624	103569	64008	Mm.335570	32204	65054	Rn.30018	15949	415402	Gga.12485	4261
**AQP 10**	89872	Hs.259048	143595	435743	-	-	-	-	-	-	-	-
**AQP 11**	282679	Hs.503345	178301	66333	Mm.29756	42797	286758	Rn.20144	13358	426725	Gga.8876	1614
												
**AQP 12**	375318	Hs.437167	184945	208760	Mm.235537	45091	367316	Rn.20532	4452	424861	Gga.19694	6436

*Ensembl Identifiers obtained had a total of 11 numerals with the appropriate number of zeros as padding. Furthermore, the identifiers were preceded by ENSG, ENSMUSG, ENSRNOG, and ENSGAL are for human, mouse, rat and chicken genes respectively. The isoform of Human AQP12 used for analysis was Aquaporin 12B.

**Table 2:** Aquaporin isoforms examined for each aquaporin type and key observations from comparative sequence analysis*

AQP Type	Organism				Observations from sequence alignment
	Human	Mouse	Rat	Chicken	
AQP1	2	1	1	1	Human transcript ENST00000265298 lacked the first 94 amino acids of ENST00000311813 which is present in the AQP1 from mouse, rat and chicken. This N-terminal region of the sequence contained the first NPA motif. However, the second NPA motif starting at position 192 in ENST00000311813 is conserved in all the 5 protein sequences
AQP3	3	1	1	1	Human transcript ENST00000379492 lacked the first 34 amino acids present in the other isoforms.
AQP4	3	2	2	2	All the organisms had multiple transcripts.
AQP5	1	2	2	3	The first NPA motif was observed in all the protein sequences. Two chicken transcripts ENSGALT00000041244 and ENSGALT00000041246 and two rodent transcripts ENSMUST00000088200 and ENSRNOT00000040874 produced isoforms that lacked the second NPA motif.
AQP7	4	3	1	1	All nine protein sequences of AQP7 contained atypical water transport NPA motifs. The first motif is NAA while the second motif was NPS.
AQP8	1	2	1	1	The first water-transport motif for chicken AQP8 was NPV
AQP9	1	4	1	2	Except for mouse transcript ENSMUST00000113569, all other AQP9 protein sequences had the second NPA motif.
AQP11	1	2	2	1	The first water transporting motif was atypical (NPC) in all the isoforms examined. Only three protein isoforms from transcripts ENSRNOT00000018091, ENSMUST00000084986 and ENST00000313578 contained the second NPA motif.
AQP12	2	1	1	1	Human transcript ENST00000373309 lacked the first NPA-like motif (NPT) present in the other sequences.

*Additional File 1 contains the multiple sequence alignment for AQP0 to AQP12. Ensembl Identifiers preceded by ENST, ENSMUST, ENSRNOT, and ENSGALT are for human, mouse, rat and chicken transcripts respectively.

**Table 3:** Prioritize aquaporin isoforms for investigation for roles other than water-transport

Aquaporin	Organism	Ensembl ID	Missing NPA or NPA-like motif
AQP1	Human	ENST00000265298	First
AQP3	Human	ENST00000343952	Second
AQP3	Human	ENST00000379492	Second
AQP4	Human	ENST00000339532	First
AQP4	Human	ENST00000383170	Second
AQP5	Mouse	ENSMUST00000088200	Second
AQP5	Rat	ENSRNOT00000040874	Second
AQP5	Chicken	ENSGALT00000041244	Second
AQP5	Chicken	ENSGALT00000041246	Second
AQP9	Mouse	ENSMUST00000113569	Second
AQP12	Human	ENST00000373309	First

**Figure 2:**
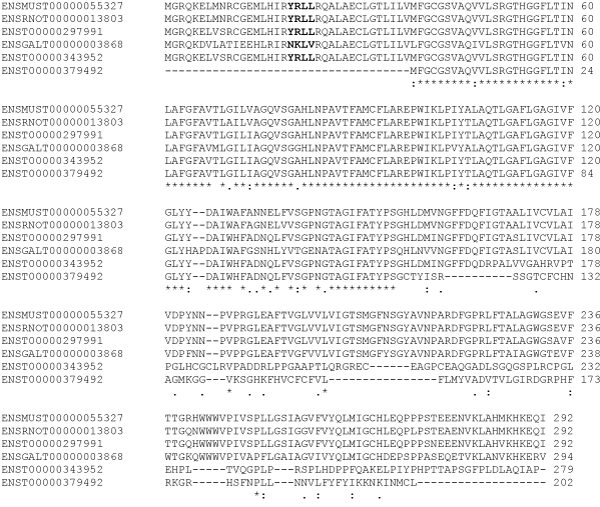
**ClustalW multiple sequence alignment of AQP3 protein isoforms predicted by the Ensembl software system**. Position 19 to 22 contains the YRLL motif for basolateral targeting. This motif is replaced in chicken AQP3 with NKLV.

**Figure 3:**
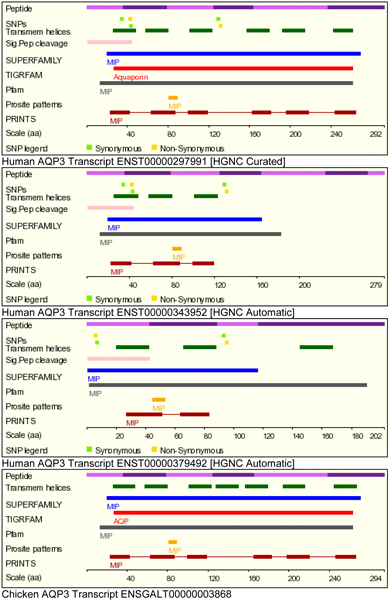
**Impact of alternative transcription and speciation on protein features of AQP3 isoforms**. There are differences in the predicted protein features of AQP3 isoforms obtained from human and chicken gene loci. In the chicken homolog, no signal peptide cleavage predicted. Images were obtained from Ensembl gene information pages. HGNC is abbreviation for Human Genome Nomenclature Committee.

**Figure 4:**
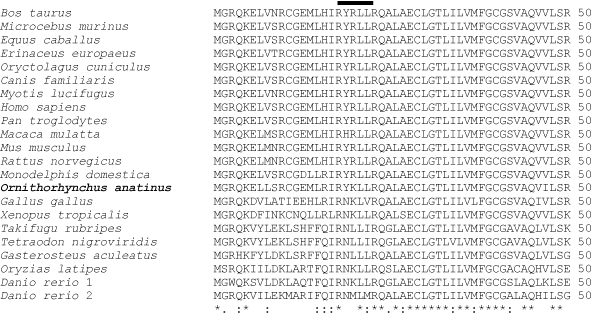
**Multiple sequence alignment of AQP3 sequences from selected vertebrates**. The platypus (*Ornithorhynchus anatinus*), a beaked mammal whose females lay eggs, had an YKLL motif aligned to the NKLV motif of the chicken sequence.

Three human transcripts were retrieved for AQP3 and one each for mouse, rat and chicken from the Ensembl (Figure 2). The protein isoform from human AQP3 transcript ENST00000379492 lacked the first 34 amino acids present in the other isoforms. This region contained the YRLL motif known to be important for basolateral sorting in epithelial cells [22-24]. The YRLL motif was replaced by a NKLV motif in the only chicken isoform obtained from transcript ENSGALT00000003868. The first NPA motif was conserved in all the AQP3 isoforms while the second NPA motif was absent in two human AQP3 isoform sequences (ENST00000343952 and ENST00000379492). The impact of alternative transcription and speciation is illustrated by protein features predicted for the three human AQP3 transcripts and one chicken AQP transcript (Figure 3). Note the difference in number of transmembrane helices predicted for the human transcripts. Interestingly, the chicken transcript lacks a prediction for signal peptide. This observation led us to further analyze the Ensembl generated pairwise sequence alignments of the chicken AQP3 with other vertebrate AQP3 showing 1-to-1 ortholog prediction. The AQP3 (ENSOANG00000014661) of the platypus (*Ornithorhynchus anatinus*) had a YKLL motif aligned to the NKLV motif of the chicken sequence (Figure 4).

**Figure 5:**
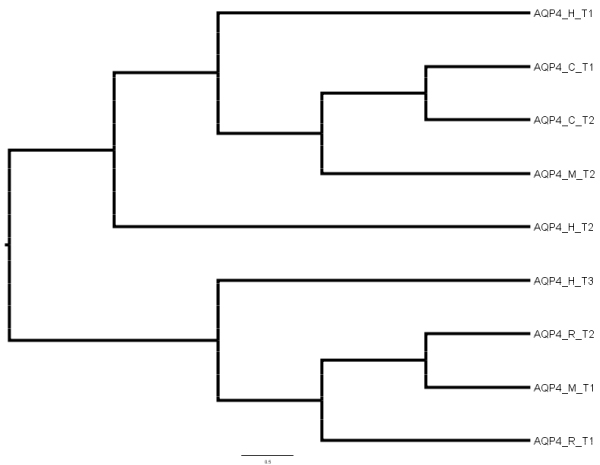
**Phylogenetic tree of AQP4 protein sequences from human (H), mouse (M), rat (R) and chicken (C)**. The symbols and their corresponding Ensembl transcripts in brackets are: AQP4_C_T1 (ENSGALT00000024413); AQP4_C_T2 (ENSGALT00000036809); AQP4_M_T1 (ENSMUST00000079081); AQP4_M_T2 (ENSMUST00000115856); AQP4_R_T1 (ENSRNOT00000021961); AQP4_R_T2 (ENSRNOT00000048109); AQP4_H_T1 (ENST00000339532); AQP4_H_T2 (ENST00000383168); AQP4_H_T3 (ENST00000383170).

AQP4 was the only aquaporin type in which the four organisms had at least two transcripts. Multiple sequence alignment of the amino acid sequences from the 9 transcripts and construction of phylogenetic tree revealed two classes of transcripts (Figure 5). The two rat transcripts (ENSRNOT00000048109 and ENSRNOT00000021961) clustered with a human transcript (ENST00000383170) and a mouse transcript (ENSMUST00000079081). In the case of the remaining 5 AQP4 transcripts, the chicken and mouse protein sequences were clustered with human transcript ENST00000339532.

In the case of AQP11, the first water transporting motif was atypical (NPC) in all the isoforms examined. Only three protein isoforms from transcripts ENSRNOT00000018091, ENSMUST00000084986 and ENST00000313578 contained the second NPA motif. AQP11 sequences had a high number of cysteine residues compared to other aquaporin types including a triplet CCC present in the N-terminal of protein sequences produced by transcripts ENSMUST00000055379, ENSMUST00000084986 and ENST00000313578. The two rat sequences had a CYC motif while chicken had a CAC motif. Cysteine residues are known to interact with sulfhydryl-reactive metals such as mercury, cadmium, lead, and arsenic [25]. A total of five transcripts for AQP12 were retrieved for the four species. The protein sequence for one of the human transcripts (ENST00000373309) lacked the first NPA-like motif (NPT) present in the other sequences. All contained the second NPA motif.

### Body site expression profiles for human, mouse, rat and chicken aquaporins

**Figure 6:**
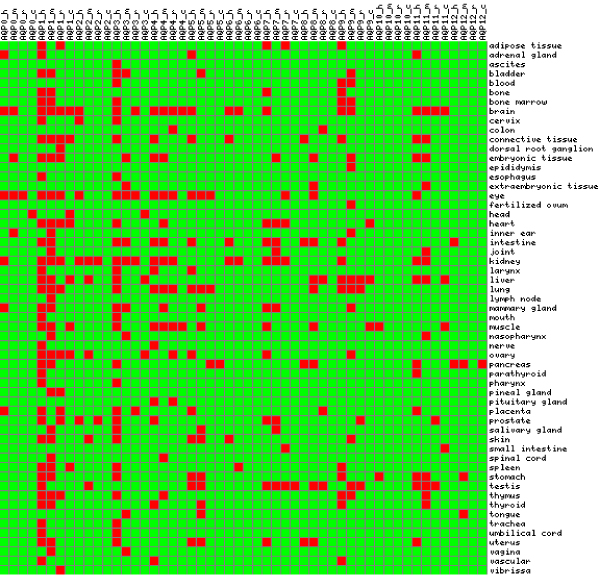
**Visualization of comparison of suggested body site expression of UniGene data for human (h), mouse (m), rat (r), and chicken (c) aquaporins**. Each aquaporin type is represented by 4 boxes corresponding to the four-digit binary number. Red box indicate presence of expression (1) while green box indicate absence of expression (0).

A total of 44 UniGene aquaporin entries from human (13), mouse (12), rat (10) and chicken (9) were identified has having suggested expression in at least one of 57 body sites based on Expressed Sequence Tags (EST) counts (Table 4). A total of 51 binary signatures described the expression of aquaporins from the organisms compared. The visualized matrix of signatures is presented in Figure 6. Furthermore, 11 body sites (brain, connective tissue, head, heart, liver, muscle, ovary, pancreas, small intestine, spleen and testis) were identified in which both chicken and mammalian aquaporins were expressed. The brain had the highest count for aquaporin expression. All four organisms expressed AQP1, AQP4 and AQP11 in the brain. There was evidence of expression of AQP12 in the intestine, pancreas, stomach, and tongue as well as expression in the pancreas for all species except rat (Figure 6, Additional file 2). We mapped chicken aquaporins to body sites in order to prioritize them for further functional analysis (Table 5).

**Table 4:** Controlled UniGene body site terms associated with expressed aquaporins from human, mouse, rat and chicken

Body Site	AQP Count	Body Site	AQP Count	Body Site	AQP Count	Body Site	AQP Count
Adipose tissue	5	Extraembryonic tissue	3	Muscle	14	Spleen	6
Adrenal gland	4	Eye	19	Nasopharynx	3	Stomach	10
Ascites	1	Fertilized ovum	1	Nerve	2	Testis	15
Bladder	6	Head	3	Ovary	10	Thymus	8
Blood	3	Heart	10	Pancreas	11	Thyroid	6
Bone	4	Inner ear	3	Parathyroid	2	Tongue	3
Bone marrow	5	Intestine	13	Pharynx	2	Trachea	2
Brain	23	Joint	3	Pineal gland	2	Umbilical cord	2
Cervix	3	Kidney	21	Pituitary gland	2	Uterus	9
Colon	2	Larynx	4	Placenta	8	Vagina	2
Connective tissue	12	Liver	15	Prostate	10	Vascular	3
Dorsal root ganglion	1	Lung	14	Salivary gland	4	Vibrissa	1
Embryonic tissue	12	Lymph node	1	Skin	8		
Epididymis	1	Mammary gland	10	Small intestine	2		
Esophagus	2	Mouth	2	Spinal cord	2		

**Table 5:** Body sites and corresponding expressed chicken aquaporins that could be used as models for understanding mammalian aquaporins

Aquaporin Type	Chicken UniGene ID	Body Site
AQP0	Gga.67	Head
AQP1	Gga.2680	Brain, Connective Tissue, Head, Heart, Liver, Muscle, Ovary, Spleen
AQP3	Gga.23800	Head, Liver, Ovary
AQP4	Gga.11374	Brain, Muscle
AQP5	Gga.6412	Pancreas
AQP7	Gga.21944	Testis
AQP9	Gga.12485	Heart, Liver, Muscle
AQP11	Gga.8876	Brain, Liver, Muscle, Small Intestine
AQP12	Gga.19694	Pancreas

## Discussion

Aquaporin cell surface proteins are emerging as important proteins beyond water transport [2,11,13,26]. However, the molecular, cellular and evolutionary basis for these novel roles are not completely known. Our high-throughput comparative sequence analysis revealed the impact of alternative transcription and speciation on the conservation of functional motifs in human, mouse, rat and chicken aquaporins. Furthermore, by using a controlled vocabulary of terms describing body sites in the UniGene database, an integrated view of suggested tissue expression of aquaporins in these four organisms was generated (Figure 6). We were unable to retrieve information from the Ensembl database on chicken AQP2 and AQP0 (Entrez Gene ID: 374124) although chicken AQP0 (MIP) has been studied in the context of early development of lens fiber and its association with gap junction proteins [16]. The predicted chicken AQP6 was annotated as AQP2-like. The annotation of AQP0, AQP2 and AQP6 from the chicken genome warrants further investigation.

Alternate splicing of mRNA as a means of generating protein diversity can occur by exon skipping [27]. All the 76 aquaporins transcripts analyzed were multi-exon transcripts and could be subject to alternative splicing with impact on protein function. For the nine aquaporin types compared, our sequence alignments uncovered aquaporin isoforms that lacked one of the two water-transport motifs (Table 3). We hypothesize that isoforms lacking one of the two NPA or NPA-like motifs characteristic will have impaired or abolished water transport function. They are also candidates for investigating roles other than water function. Another mechanism for variation that can impact function of aquaporins is single nucleotide polymorphisms (SNPs). Previous work has demonstrated that novel genetic variants of AQP4 resulting from single nucleotide polymorphisms (SNP) showed reduced water permeability [28]. We observed from the Ensembl database (Release 50 July 2008) that a total of 19 reference SNPs were mapped to the following chicken aquaporins: AQP1, AQP7, AQP8, AQP9 and AQP12. As the chicken genome become further characterized for polymorphisms, it may become possible to gain insights into impact of SNP variation on aquaporin function and organism phenotype.

Most proteins that target the plasma membrane contain signals within their cytoplasmic termini that permit their recruitment into endocytic vesicles, which in turn facilitates their selective compartmentalization in the apical or basolateral membranes selectively [23]. We were interested in identifying avian protein isoforms in which the functional motifs were different from mammalian aquaporin isoforms. We have previously investigated the compartmentalization of AQP3 and AQP10 in the human intestine in which we observed that the basolateral sorting motif "YRLL" is present in AQP3 but absent in AQP10 [22]. Based on the UniGene suggested expression profiles there was no suggestion for the expression of chicken AQP3 in the intestine. Furthermore, the signal peptide for targeting was not predicted for chicken AQP3 in the Ensembl resource (Figure 3). Tissue expression for this chicken AQP3 was suggested for head, liver, and ovary. Comparative sequence analysis provided evidence that chicken AQP3 lacks the YRLL motif (NKLV was observed) suggesting that it may not be targeted to the basolateral membrane of the intestine. The multiple sequence alignment of the AQP3 protein sequences from the four organisms revealed that the amino acids immediately before and after the YRLL and NKLV motifs were conserved (Figure 2).

According to the ProTeus (PROtein TErminUS) tool [29], the NKLV protein signature is a short linear significant signature in termini of proteins with a corresponding Gene Ontology Cellular Component of "extracellular". Based on alignments available at the Ensembl resource [21] for AQP3 from fish (*Danio rerio*, *Oryzias latipes*, *Takifugu rubripes*, and *Tetraodon nigroviridis*) and an amphibian (*Xenopus tropicalis*), the ancestral motifs of the sorting motifs found in human may be NKLL or NMLM (Figure 4). Thus, the presence of the amino acid tyrosine (Y) in this sorting tetrapeptide in the platypus suggests the point in the evolution of AQP3 where the asparagine residue was replaced by the critical tyrosine required for sorting function [30]. The genome of *O. anatinus *has been proposed has useful for informing human genome sequence and critical link to understanding the differences between avian and mammalian genomes [31,32]. These observations provide novel evolutionarily insights into the localization signals encoded in the termini of AQP3.

We have used a binary encoding integration strategy to gain a comprehensive view of suggested tissue expression of aquaporins for four organisms. Higher-order patterns in a binary vector space that encodes the presence (1) or absence (0) of feature of interest is an approach for integrating genome-wide numerical datasets [33-35]. Several advantages offered by the binary integration of high-throughput gene expression data include computational efficiency and noise resilience [35]. Our matrix revealed that AQP4, in addition to AQP1 and AQP11 were expressed in the brain of all the four organisms. These three aquaporin types are candidates for comparative experimental investigation of aquaporin in brain function. In addition, the analysis delivered a set of body sites in which there was evidence of expression of at least one mammalian aquaporin and chicken aquaporin.

The functions of AQP11 and AQP12 in vertebrate physiology are not completely understood [1,3]. We observed that AQP11 had the highest number of cysteine residues compared to other aquaporins. AQP11 is expressed in the Purkinje cells of the brain cerebellar [1], a site that have been implicated in the pathophysiology of autism [36,37]. Exposure of chick embryo to the environmental metal pollutant methylmercury led to reduction in the number of Purkinje cells [38]. Furthermore, there were adverse post-natal behavioral, morphological and biochemical consequences. Mercury ions are known to regulate aquaporin function by interacting with cysteine residues [39,40]. Interestingly, the first pore forming motif of AQP11 found in Loop B has a motif of NPC instead of the NPA. However, the second motif is NPA consistent with other AQPs. AQP11 is the only human aquaporin with the tri-cysteine (CCC) motif. These observations led us to hypothesize that AQP11 may be a target in the pathophysiology of neurodegenerative disorders like autism. We are currently studying the specificity and affinity of a range of cations to further understand the interaction of cations with aquaporins in the function of the central nervous system. With the availability of the chicken genome, the chick embryo has the potential to serve as an important model for the study of the development of neurodegenerative disorders [38,41].

In the case of AQP12, there was suggested expression in the pancreas of human, mouse and chicken (Table 4) with chicken having the highest expression level. AQP12 is localized intracellularly in the pancreatic acinar cells, the site for synthesis of digestive enzymes [3]. Histopathological studies have confirmed that the highly pathogenic H5N1 virus that causes avian influenza in chicken and other avian species causes multifocal necrosis in the pancreatic acinar cells suggesting that the effect of avian influenza on the function of AQP12 warrants further investigation.

## Conclusion

This study demonstrates that the chicken genome combined with a comprehensive controlled vocabulary-facilitated integration of UniGene suggested body site expression can drive generation of hypotheses related to the function of avian and mammalian aquaporins. We describe the generation of hypotheses related to (i) the impact of alternative transcription and speciation on the conservation of functional motifs in human, mouse, rat and chicken aquaporins; (ii) identification of the emergence of basolateral targeting in mammalian species (iii) possibility of cysteine-rich AQP11 as target in the pathophysiology of neurodegenerative disorders; and (iv) impact on the function of pancreas-expressed AQP12 during pancreatotropic necrosis associated with avian influenza virus infection.

## Methods

### Comparison of predicted aquaporin transcripts from human, mouse, rat and chicken

Predictions of genes encoding aquaporins from human, mouse, rat and chicken genomes were obtained from the Ensembl project (Release 50 July 2008) [21] and the Entrez Gene database at the National Center for Biotechnology Information (NCBI) [20]. In both databases, the human aquaporin information was used as the starting point to extract predicted homologous proteins in the other three species. The amino acid sequences encoding predicted transcripts associated with aquaporin genes in which both an Ensembl and Entrez Gene record exist were further compared. We sought to determine the conservation of functional motifs among predicted transcripts from orthologous genes. Therefore, the amino acid sequence diversity encoded by aquaporin transcripts was determined by multiple sequence alignment of orthologs using ClustalW. In order to visualize the relationship of selected sequences, neighbor-joining tree bootstrapped using 1,000 random samples of sites from the alignment was constructed using the ClustalW software at the DNA Data Bank of Japan [42]. FigTree software version 1.12 [43] was used to view the phylogenetic trees.

### UniGene expression profiles for human, mouse, rat and chicken aquaporins

Each NCBI UniGene Cluster contains set of transcript sequences that appear to come from the same transcription locus (gene or expressed pseudogene) as well as other information including expression profile in body sites and developmental stages [20]. The UniGene cluster identifier for each of the aquaporins was extracted from the Entrez Gene record and verified manually. In instances where more than one UniGene entry was associated the Entrez Gene, the unambiguous UniGene entry was selected. The value of the Transcript per million (TPM) for each body site was programmatically extracted from the UniGene Expression Profile Viewer page.

There was need to identify chicken and mammalian aquaporins that were expressed in the same body site based on controlled vocabulary term used in UniGene. Therefore, a 4-digit binary signature was constructed to encode the presence or absence of species aquaporin expression. Thus a matrix consisting of 52-digit binary signature and the number of body sites was constructed and visualized using matrix2png [44].

## Competing interests

The authors declare that they have no competing interests.

## Authors' contributions

RDI, RVR and HHPC conceived and designed the study. RDI wrote scripts to retrieve compare and integrate datasets and drafted the manuscript. RVR carried out sequence alignment and motif analysis and helped to draft the manuscript. CDJ assembled the aquaporin datasets from multiple organisms, evaluated binary matrix integration and helped to draft the manuscript. TOO developed scripts and computational pipelines to facilitate literature extraction, search and visualization of binary matrices; and helped to draft the manuscript. HHPC helped to interpret the study results, coordinated the study and helped to draft the manuscript. All authors read and approved the final manuscript.

## Supplementary Material

Additional file 1 **Multiple sequence alignment of aquaporins isoforms from human, mouse, rat and chicken**. ClustalW multiple sequence alignment of aquaporin types from human, mouse, rat and chicken. Identifiers preceded by ENST, ENSMUST, ENSRNOT, and ENSGALT are for human, mouse, rat and chicken transcripts respectively. Click here for file

Additional file 2 UniGene Transcripts per million and spot intensity of human, mouse and chicken AQP12 expressed in pancreas. Click here for file
