# Supplementary material for: Integrative sequence and tissue expression profiling of chicken and mammalian aquaporins
Source: BMC Genomics. 2009 Jul 14;10(Suppl 2):S7. doi: 10.1186/1471-2164-10-S2-S7 (PMC2966333; doi:10.1186/1471-2164-10-S2-S7)
Supplement: Additional file 1 — Multiple sequence alignment of aquaporins isoforms from human, mouse, rat and chicken. ClustalW multiple sequence alignment of aquaporin types from human, mouse, rat and chicken. Identifiers preceded by ENST, ENSMUST, ENSRNOT, and ENSGALT are for human, mouse, rat and chicken transcripts respectively. [file 1471-2164-10-S2-S7-S1.pdf]

AQP0 - CLUSTAL W (1.83) multiple sequence alignment

```
ENSMUST00000026455      MWELRSASFWRAIFAEFFATLFYVFFGLGASLRWAPGPLHVLQVALAFGLALATLVQTVG 60
ENSRNOT00000004223      MWELRSASFWRAIFAEFFATLFYVFFGLGSSLRWAPGPLHVLQVALAFGLALATLVQTVG 60
ENST00000257979         MWELRSASFWRAIFAEFFATLFYVFFGLGSSLRWAPGPLHVLQVAMAFGLALATLVQSVG 60
                        *****************************:***************:***********:**

ENSMUST00000026455      HISGAHVNPAVTFAFLVGSQMSLLRAFCYIAAQLLGAVAGAAVLYSVTPPAVRGNLALNT 120
ENSRNOT00000004223      HISGAHVNPAVTFAFLVGSQMSLLRAFCYIAAQLLGAVAGAAVLYSVTPPAVRGNLALNT 120
ENST00000257979         HISGAHVNPAVTFAFLVGSQMSLLRAFCYMAAQLLGAVAGAAVLYSVTPPAVRGNLALNT 120
                        *****************************:******************************

ENSMUST00000026455      LHAGVSVGQATTVEIFLTLQFVLCIFATYDERRNGRMGSVALAVGFSLTLGHLFGMYYTG 180
ENSRNOT00000004223      LHAGVSVGQATTVEIFLTLQFVLCIFATYDERRNGRMGSVALAVGFSLTLGHLFGMYYTG 180
ENST00000257979         LHPAVSVGQATTVEIFLTLQFVLCIFATYDERRNGQLGSVALAVGFSLALGHLFGMYYTG 180
                        **..********************************::**********:***********

ENSMUST00000026455      AGMNPARSFAPAILTRNFSNHWVYWVGPIIGGGLGSLLYDFLLFPRLKSVSERLSILKGA 240
ENSRNOT00000004223      AGMNPARSFAPAILTRNFSNHWVYWVGPIIGGGLGSLLYDFLLFPRLKSVSERLSILKGA 240
ENST00000257979         AGMNPARSFAPAILTGNFTNHWVYWVGPIIGGGLGSLLYDFLLFPRLKSISERLSVLKGA 240
                        *************** **:******************************:*****:****

ENSMUST00000026455      RPSDSNGQPEGTGEPVELKTQAL 263
ENSRNOT00000004223      RPSDSNGQPEGTGEPVELKTQAL 263
ENST00000257979         KPDVSNGQPEVTGEPVELNTQAL 263
                        :*. ****** *******:****
```

AQP1 - CLUSTAL W (1.83) multiple sequence alignment

```
ENSMUST00000004774      MASEIKKKLFWRAVVAEFLAMTLFVFISIGSALGFNYPLERN-QTLVQDNVKVSLAFGLS 59
ENSRNOT00000015692      MASEIKKKLFWRAVVAEFLAMTLFVFISIGSALGFNYPLERN-QTLVQDNVKVSLAFGLS 59
ENSGALT00000008364      MASEFKKKMFWRAVVAEFLAMILFIFISIGSALGFNFPVSVNGTSATQDNVKVSLAFGLS 60
ENST00000311813         MASEFKKKLFWRAVVAEFLATTLFVFISIGSALGFKYPVGNN-QTAVQDNVKVSLAFGLS 59
ENST00000265298         ------------------------------------------------------------


ENSMUST00000004774      IATLAQSVGHISGAHLNPAVTLGLLLSCQISILRAVMYIIAQCVGAIVATAILSGITSSL 119
ENSRNOT00000015692      IATLAQSVGHISGAHLNPAVTLGLLLSCQISILRAVMYIIAQCVGAIVASAILSGITSSL 119
ENSGALT00000008364      IATMAQSVGHISGAHLNPAVTLGLLLSCQISIFKALMYILAQCLGAVVATAILSGVTSSL 120
ENST00000311813         IATLAQSVGHISGAHLNPAVTLGLLLSCQISIFRALMYIIAQCVGAIVATAILSGITSSL 119
ENST00000265298         ------------------------------------MYIIAQCVGAIVATAILSGITSSL 24
                                                            ***:***:**:**:*****:****

ENSMUST00000004774      VDNSLGRNDLAHGVNSGQGLGIEIIGTLQLVLCVLATTDRRRRDLGGSAPLAIGLSVALG 179
ENSRNOT00000015692      LENSLGRNDLARGVNSGQGLGIEIIGTLQLVLCVLATTDRRRRDLGGSAPLAIGLSVALG 179
ENSGALT00000008364      PYNSLGLNALAKGINAGQGLGIEIIATLQLVLCVLATTDRRRNDVSGSAPLAIGLSVALG 180
ENST00000311813         TGNSLGRNDLADGVNSGQGLGIEIIGTLQLVLCVLATTDRRRRDLGGSAPLAIGLSVALG 179
ENST00000265298         TGNSLGRNDLADGVNSGQGLGIEIIGTLQLVLCVLATTDRRRRDLGGSAPLAIGLSVALG 84
                          **** * ** *:*:*********.****************.*:.**************

ENSMUST00000004774      HLLAIDYTGCGINPARSFGSAVLTRNFSNHWIFWVGPFIGGALAVLIYDFILAPRSSDFT 239
ENSRNOT00000015692      HLLAIDYTGCGINPARSFGSAVLTRNFSNHWIFWVGPFIGSALAVLIYDFILAPRSSDFT 239
ENSGALT00000008364      HLLAIDYTGCGINPARSFGSALIANNFENHWIFWVGPIIGGAGAALIYDFILAPRSSDLT 240
ENST00000311813         HLLAIDYTGCGINPARSFGSAVITHNFSNHWIFWVGPFIGGALAVLIYDFILAPRSSDLT 239
ENST00000265298         HLLAIDYTGCGINPARSFGSAVITHNFSNHWIFWVGPFIGGALAVLIYDFILAPRSSDLT 144
                        *********************:::.**.*********:**.* *.*************:*

ENSMUST00000004774      DRMKVWTSGQVEEYDLDADDINSR-----------------VEMKPK------------- 269
ENSRNOT00000015692      DRMKVWTSGQVEEYDLDADDINSR-----------------VEMKPK------------- 269
ENSGALT00000008364      DRVKVWTSGQVEEYDLEGDDMNSR-----------------VEMKPK------------- 270
ENST00000311813         DRVKVWTSGQVEEYDLDADDINSR-----------------VEMKPK------------- 269
ENST00000265298         DRVKVWTSGQVEEYDLDADDINSRSFLGTKIYQFTHSLEVVEEVKERDPPASRPSEHDGR 204
                        **:*************:.**:***                  *:* :

ENSMUST00000004774      ------------------------
ENSRNOT00000015692      ------------------------
ENSGALT00000008364      ------------------------
ENST00000311813         ------------------------
ENST00000265298         CARKSPLAPKLLTDSPAQVPGILP 228
```

```
AQP2 - CLUSTAL W (1.83) multiple sequence alignment


ENSMUST00000023752      MWELRSIAFSRAVLAEFLATLLFVFFGLGSALQWASSPPSVLQIAVAFGLGIGTLVQALG 60
ENSRNOT00000000324      MWELRSIAFSRAVLAEFLATLLFVFFGLGSALQWASSPPSVLQIAVAFGLGIGTLVQALG 60
ENST00000199280         MWELRSIAFSRAVFAEFLATLLFVFFGLGSALNWPQALPSVLQIAMAFGLGIGTLVQALG 60
                        *************:*******************:*..: *******:*************

ENSMUST00000023752      HVSGAHINPAVTVACLVGCHVSFLRAAFYVAAQLLGAVAGAAILHEITPVEIRGDLAVNA 120
ENSRNOT00000000324      HVSGAHINPAVTVACLVGCHVSFLRAAFYVAAQLLGAVAGAAILHEITPVEIRGDLAVNA 120
ENST00000199280         HISGAHINPAVTVACLVGCHVSVLRAAFYVAAQLLGAVAGAALLHEITPADIRGDLAVNA 120
                        *:********************.*******************:******.:*********

ENSMUST00000023752      LHNNATAGQAVTVELFLTMQLVLCIFASTDERRSDNLGSPALSIGFSVTLGHLLGIYFTG 180
ENSRNOT00000000324      LHNNATAGQAVTVELFLTMQLVLCIFASTDERRGDNLGSPALSIGFSVTLGHLLGIYFTG 180
ENST00000199280         LSNSTTAGQAVTVELFLTLQLVLCIFASTDERRGENPGTPALSIGFSVALGHLLGIHYTG 180
                        * *.:*************:**************.:* *:*********:*******::**

ENSMUST00000023752      CSMNPARSLAPAVVTGKFDDHWVFWIGPLVGAVIGSLLYNYLLFPSTKSLQERLAVLKGL 240
ENSRNOT00000000324      CSMNPARSLAPAVVTGKFDDHWVFWIGPLVGAIIGSLLYNYLLFPSAKSLQERLAVLKGL 240
ENST00000199280         CSMNPARSLAPAVVTGKFDDHWVFWIGPLVGAILGSLLYNYVLFPPAKSLSERLAVLKGL 240
                        ********************************::*******:***.:***.*********

ENSMUST00000023752      EPDTDWEEREVRRRQSVELHSPQSLPRGSKA 271
ENSRNOT00000000324      EPDTDWEEREVRRRQSVELHSPQSLPRGSKA 271
ENST00000199280         EPDTDWEEREVRRRQSVELHSPQSLPRGTKA 271
                        ****************************:**
```

AQP3 - CLUSTAL W (1.83) multiple sequence alignment

```
ENSMUST00000055327      MGRQKELMNRCGEMLHIRYRLLRQALAECLGTLILVMFGCGSVAQVVLSRGTHGGFLTIN 60
ENSRNOT00000013803      MGRQKELMNRCGEMLHIRYRLLRQALAECLGTLILVMFGCGSVAQVVLSRGTHGGFLTIN 60
ENST00000297991         MGRQKELVSRCGEMLHIRYRLLRQALAECLGTLILVMFGCGSVAQVVLSRGTHGGFLTIN 60
ENSGALT00000003868      MGRQKDVLATIEEHLRIRNKLVRQALAECLGTLILVLFGCGSVAQIVLSRGTHGGFLTVN 60
ENST00000343952         MGRQKELVSRCGEMLHIRYRLLRQALAECLGTLILVMFGCGSVAQVVLSRGTHGGFLTIN 60
ENST00000379492         ------------------------------------MFGCGSVAQVVLSRGTHGGFLTIN 24
                                                            :********:************:*

ENSMUST00000055327      LAFGFAVTLGILVAGQVSGAHLNPAVTFAMCFLAREPWIKLPIYALAQTLGAFLGAGIVF 120
ENSRNOT00000013803      LAFGFAVTLAILVAGQVSGAHLNPAVTFAMCFLAREPWIKLPIYTLAQTLGAFLGAGIVF 120
ENST00000297991         LAFGFAVTLGILIAGQVSGAHLNPAVTFAMCFLAREPWIKLPIYTLAQTLGAFLGAGIVF 120
ENSGALT00000003868      LAFGFAVMLGILIAGQVSGGHLNPAVTFAMCFLAREPWIKLPVYALAQTLGAFLGAGIVF 120
ENST00000343952         LAFGFAVTLGILIAGQVSGAHLNPAVTFAMCFLAREPWIKLPIYTLAQTLGAFLGAGIVF 120
ENST00000379492         LAFGFAVTLGILIAGQVSGAHLNPAVTFAMCFLAREPWIKLPIYTLAQTLGAFLGAGIVF 84
                        ******* *.**:******.**********************:*:***************

ENSMUST00000055327      GLYY--DAIWAFANNELFVSGPNGTAGIFATYPSGHLDMVNGFFDQFIGTAALIVCVLAI 178
ENSRNOT00000013803      GLYY--DAIWAFAGNELVVSGPNGTAGIFATYPSGHLDMVNGFFDQFIGTAALIVCVLAI 178
ENST00000297991         GLYY--DAIWHFADNQLFVSGPNGTAGIFATYPSGHLDMINGFFDQFIGTASLIVCVLAI 178
ENSGALT00000003868      GLYHAPDAIWAFGSNHLYVTGENATAGIFATYPSQHLNVVNGFFDQFIGTASLIVCVLAI 180
ENST00000343952         GLYY--DAIWHFADNQLFVSGPNGTAGIFATYPSGHLDMINGFFDQDRPALVVGAHRVPT 178
ENST00000379492         GLYY--DAIWHFADNQLFVSGPNGTAGIFATYPSGCTYISR----------SSGTCFCHN 132
                        ***:  **** *..*.* *:* *.**********    : .             .

ENSMUST00000055327      VDPYNN--PVPRGLEAFTVGLVVLVIGTSMGFNSGYAVNPARDFGPRLFTALAGWGSEVF 236
ENSRNOT00000013803      VDPYNN--PVPRGLEAFTVGLVVLVIGTSMGFNSGYAVNPARDFGPRLFTALAGWGSEVF 236
ENST00000297991         VDPYNN--PVPRGLEAFTVGLVVLVIGTSMGFNSGYAVNPARDFGPRLFTALAGWGSAVF 236
ENSGALT00000003868      VDPFNN--PVPPGLEAFTVGFVVLVIGTSMGFYSGYAVNPARDFGPRLFTAIAGWGTEVF 238
ENST00000343952         PGLHCGCLRVPADDRLPPGAAPTLQRGREC------EAGPCEAQGADLSGQGSPLRCPGL 232
ENST00000379492         AGMKGG---VKSGHKFHVCFCFVL----------------FLMYVADVTLGIRDGRPHF 173
                         .   .   *  . .       .*                     . :           :

ENSMUST00000055327      TTGRHWWWVPIVSPLLGSIAGVFVYQLMIGCHLEQPPPSTEEENVKLAHMKHKEQI 292
ENSRNOT00000013803      TTGQNWWWVPIVSPLLGSIGGVFVYQLMIGCHLEQPLPSTEAENVKLAHMKHKEQI 292
ENST00000297991         TTGQHWWWVPIVSPLLGSIAGVFVYQLMIGCHLEQPPPSNEEENVKLAHVKHKEQI 292
ENSGALT00000003868      WTGKQWWWVPIVAPFLGAIAGVIVYQLMIGCHDEPSPPASEQETVKLANVKHKERV 294
ENST00000343952         EHPL-----TVQGPLP---RSPLHDPPFQAKELPIYPHPTTAPSGFPLDLAQIAP- 279
ENST00000379492         RKGR-----HSFNPLL---NNVLFYFYIKKNKINMCL------------------ 202
                                     *:     . :    :   .
```

AQP4 - CLUSTAL W (1.83) multiple sequence alignment

```
ENST00000339532         ------------------------------------------------------------
ENST00000383168         ------------------------------------------------------------
ENSGALT00000024413      ------------------------------------------SKCARLCKCESIMVAFKG 18
ENSGALT00000036809      ------------------------------------------------------------
ENSMUST00000115856      MVHGFGCFVFFFLISLSSLWASEDSTCNSTLPLCHLATTLDCCKCGHSCSRESIMVAFKG 60
ENSMUST00000079081      --------------------------------MSDRAAARRWGKCGHSCSRESIMVAFKG 28
ENST00000383170         ------------------------------------------------------------
ENSRNOT00000048109      ------------------------------------------------------------
ENSRNOT00000021961      ------------------------------------------------------------


ENST00000339532         ------------------------------------------------------------
ENST00000383168         ------------------------------------------------------------
ENSGALT00000024413      VWTQPFWKAVSAEFLAMLIFVLLSLGSTINWGGSEKPLPVDMVLISLCFGLSIATMVQCF 78
ENSGALT00000036809      ------------------------------------------------------------
ENSMUST00000115856      VWTQAFWKAVSAEFLATLIFVLLGVGSTINWGGSENPLPVDMVLISLCFGLSIATMVQCF 120
ENSMUST00000079081      VWTQAFWKAVSAEFLATLIFVLLGVGSTINWGGSENPLPVDMVLISLCFGLSIATMVQCF 88
ENST00000383170         ------------------------------------------------------------
ENSRNOT00000048109      ------------------------------------------------------------
ENSRNOT00000021961      ------------------------------------------------------------


ENST00000339532         ----------------MSDRPTARRWGKCGPLCTREN----------------------- 21
ENST00000383168         ----------------MSDRPTARRWGKCGPLCTRENIMVAFKGVWTQAFWKAVTAEFLA 44
ENSGALT00000024413      GHISGGHINPAVTVAMVCTRKISLAKSVFYILAQCLG----------------------- 115
ENSGALT00000036809      ------HINPAVTVAMVCTRKISLAKSVFYILAQCLG----------------------- 31
ENSMUST00000115856      GHISGGHINPAVTVAMVCTRKISIAKSVFYIIAQCLG----------------------- 157
ENSMUST00000079081      GHISGGHINPAVTVAMVCTRKISIAKSVFYIIAQCLG----------------------- 125
ENST00000383170         ---------CTLAGEGMSDRPTARRWGKCGPLCTREN----------------------- 28
ENSRNOT00000048109      ----------------MSDGAAARRWGKCGPPCSRES----------------------- 21
ENSRNOT00000021961      ----------------MSDGAAARRWGKCGPPCSRES----------------------- 21
                                        :.    :   .     .   .

ENST00000339532         ------------------------------------------------------------
ENST00000383168         MLIFVLLSLGSTINWGGTEKPLPVDMVLISLCFGLSIATMVQCFGHISGGHINPAVTVAM 104
ENSGALT00000024413      ------------------------------------------------------------
ENSGALT00000036809      ------------------------------------------------------------
ENSMUST00000115856      ------------------------------------------------------------
ENSMUST00000079081      ------------------------------------------------------------
ENST00000383170         ------------------------------------------------------------
ENSRNOT00000048109      ------------------------------------------------------------
ENSRNOT00000021961      ------------------------------------------------------------


ENST00000339532         ----------------------IMGAGILYLVTPPSVVGGLGVTMVHGNLTAG------- 52
ENST00000383168         VCTRKISIAKSVFYIAAQCLGAIIGAGILYLVTPPSVVGGLGVTMVHGNLTAG------- 157
ENSGALT00000024413      ---------------------AIVGAGILYLITPPSVVGGLGVTAVHGDLSAG------- 147
ENSGALT00000036809      ---------------------AIVGAGILYLITPPSVVGGLGVTAVHGDLSAG------- 63
ENSMUST00000115856      ---------------------AIIGAGILYLVTPPSVVGGLGVTTVHGNLTAG------- 189
ENSMUST00000079081      ---------------------AIIGAGILYLVTPPSVVGGLGVTTVHGNLTAG------- 157
ENST00000383170         --------------------IMVAFKGVWTQAFWKAVTAEFLAMLIFVLLSLGSTINWGG 68
ENSRNOT00000048109      --------------------IMVAFKGVWTQAFWKAVTAEFLAMLIFVLLSVGSTINWGG 61
ENSRNOT00000021961      --------------------IMVAFKGVWTQAFWKAVTAEFLAMLIFVLLSVGSTINWGG 61
                                              :   *:       :*.. : .  :.  *: *

ENST00000339532         --HGLLVELIIT---FQLVFTIFASCDSKRT--DVTGSIALAIGFSVAIGHLFAINYTGA 105
ENST00000383168         --HGLLVELIIT---FQLVFTIFASCDSKRT--DVTGSIALAIGFSVAIGHLFAINYTGA 210
ENSGALT00000024413      --HGLLVELIIT---FQLVFTIFASCDSKRS--DVTGSVALAIGFSVAIGHLFAINYTGA 200
ENSGALT00000036809      --HGLLVELIIT---FQLVFTIFASCDSKRS--DVTGSVALAIGFSVAIGHLFAINYTGA 116
ENSMUST00000115856      --HGLLVELIIT---FQLVFTIFASCDSKRT--DVTGSIALAIGFSVAIGHLFAINYTGA 242
ENSMUST00000079081      --HGLLVELIIT---FQLVFTIFASCDSKRT--DVTGSIALAIGFSVAIGHLFAINYTGA 210
ENST00000383170         TEKPLPVDMVLISLCFGLSIATMVQCFGHISGGHINPAVTVAMVCTRKISIAKSVFYIAA 128
ENSRNOT00000048109      SENPLPVDMVLISLCFGLSIATMVQCFGHISGGHINPAVTVAMVCTRKISIAKSVFYITA 121
ENSRNOT00000021961      SENPLPVDMVLISLCFGLSIATMVQCFGHISGGHINPAVTVAMVCTRKISIAKSVFYITA 121
                          : * *::::    * * :: :..* .: :  .:. ::::*:  :  *.    :: *  *

ENST00000339532         SMNPAR------------------------------------------------------ 111
ENST00000383168         SMNPAR------------------------------------------------------ 216
ENSGALT00000024413      SMNPAR------------------------------------------------------ 206
ENSGALT00000036809      SMNPA------------------------------------------------------- 121
ENSMUST00000115856      SMNPAR------------------------------------------------------ 248
ENSMUST00000079081      SMNPAR------------------------------------------------------ 216
ENST00000383170         QCVG-------------------------------------------------------- 132
ENSRNOT00000048109      QCLGAIIGAGILYLVTPPSVVGGLGVTTIN------------------------------ 151
ENSRNOT00000021961      QCLGAIIGAGILYLVTPPSVVGGLGVTTVHGNLTAGHGLLVELIITFQLVFTIFASCDSK 181
                        .

ENST00000339532         -----------------------------------SFGPAVIMGNWENHWIYWVGPIIGA 136
ENST00000383168         -----------------------------------SFGPAVIMGNWENHWIYWVGPIIGA 241
ENSGALT00000024413      -----------------------------------SFGPAVIMGKWENQWVYWVGPIIGA 231
ENSGALT00000036809      ------------------------------------------------------------
```

```
ENSMUST00000115856      -----------------------------------SFGPAVIMGNWANHWIYWVGPIMGA 273
ENSMUST00000079081      -----------------------------------SFGPAVIMGNWANHWIYWVGPIMGA 241
ENST00000383170         -------------------------------------------------------PIIGA 137
ENSRNOT00000048109      -------------------------YTGASMNPARSFGPAVIMGNWENHWIYWVGPIIGA 186
ENSRNOT00000021961      RTDVTGSVALAIGFSVAIGHLFAINYTGASMNPARSFGPAVIMGNWENHWIYWVGPIIGA 241

ENST00000339532         VLAGGLYEYVFCPDVEFKRRFKEAFSKAAQQTKGSYMEVEDNRSQVETDDLILKPGVVHV 196
ENST00000383168         VLAGGLYEYVFCPDVEFKRRFKEAFSKAAQQTKGSYMEVEDNRSQVETDDLILKPGVVHV 301
ENSGALT00000024413      VLAGALYEYVYCPDMELKRRFKDVFSKATQPSKGKYIEVDDTRSHVETDDLILKPGIVHV 291
ENSGALT00000036809      ------------------------------------------------------------
ENSMUST00000115856      VLAGALYEYVFCPDVELKRRLKEAFSKAAQQTKGSYMEVEDNRSQVETEDLILKPGVVHV 333
ENSMUST00000079081      VLAGALYEYVFCPDVELKRRLKEAFSKAAQQTKGSYMEVEDNRSQVETEDLILKPGVVHV 301
ENST00000383170         VLAGGLYEYVFCPDVEFKRRFKEAFSKAAQQTKGSYMEVEDNRSQVETDDLILKPGVVHV 197
ENSRNOT00000048109      VLAGALYEYVFCPDVELKRRLKEAFSKAAQQTKGSYMEVEDNRSQVETEDLILKPGVVHV 246
ENSRNOT00000021961      VLAGALYEYVFCPDVELKRRLKEAFSKAAQQTKGSYMEVEDNRSQVETEDLILKPGVVHV 301

ENST00000339532         IDVDRGEEKKGKDQSGEVLSSV 218
ENST00000383168         IDVDRGEEKKGKDQSGEVLSSV 323
ENSGALT00000024413      IDIDRSEDKKGRDPSSEVLSSV 313
ENSGALT00000036809      ----------------------
ENSMUST00000115856      IDIDRGEEKKGKDSSGEVLSSV 355
ENSMUST00000079081      IDIDRGEEKKGKDSSGEVLSSV 323
ENST00000383170         IDVDRGEEKKGKDQSGEVLSSV 219
ENSRNOT00000048109      IDIDRGDEKKGKDSSGEVLSSV 268
ENSRNOT00000021961      IDIDRGDEKKGKDSSGEVLSSV 323
```

AQP5 - CLUSTAL W (1.83) multiple sequence alignment

```
ENSMUST00000042340        ----------------------------------MKKEVCSVAFFKAVFAEFLATLIFVF 26
ENSRNOT00000024102        ----------------------------------MKKEVCSLAFFKAVFAEFLATLIFVF 26
ENSMUST00000088200        ----------------------------------MKKEVCSVAFFKAVFAEFLATLIFVF 26
ENSRNOT00000040874        ----------------------------------MKKEVCSLAFFKAVFAEFLATLIFVF 26
ENST00000293599           ----------------------------------MKKEVCSVAFLKAVFAEFLATLIFVF 26
ENSGALT00000041244        -----------------------------------------------------STLIFVF 7
ENSGALT00000041246        -------------------------------------EILTLAFARAVFVEFISTLIFVF 23
ENSGALT00000004288        APPDQQRQWELSSKAVVFFAPRVHRGSHPPPTPTMKREILTLAFARAVFVEFISTLIFVF 60
                                                                               :******

ENSMUST00000042340        FGLGSALKWPSALPTILQISIAFGLAIGTLAQALGPVSGGHINPAITLALLIGNQISLLR 86
ENSRNOT00000024102        FGLGSALKWPSALPTILQISIAFGLAIGTLAQALGPVSGGHINPAITLALLIGNQISLLR 86
ENSMUST00000088200        FGLGSALKWPSALPTILQISIAFGLAIGTLAQALGPVSGGHINPAITLALLIGNQISLLR 86
ENSRNOT00000040874        FGLGSALKWPSALPTILQISIAFGLAIGTLAQALGPVSGGHINPAITLALLIGNQISLLR 86
ENST00000293599           FGLGSALKWPSALPTILQIALAFGLAIGTLAQALGPVSGGHINPAITLALLVGNQISLLR 86
ENSGALT00000041244        IGLGSALKWPSALPSILQISLAFGLAIGTLVQAFGHISGAHINPAVTIAFFVGNQISFLR 67
ENSGALT00000041246        IGLGSALKWPSALPSILQISLAFGLAIGTLVQAFGHISGAHINPAVTIAFFVGNQISFLR 83
ENSGALT00000004288        IGLGSALKWPSALPSILQISLAFGLAIGTLVQAFGHISGAHINPAVTIAFFVGNQISFLR 120
                          :*************:****::*********.**:* :**.*****:*:*:::*****:**

ENSMUST00000042340        AIFYVAAQLVGAIAGAGILYWLAPGNARGNLAVN---ALSNNTTPGKAVVVELILTFQLA 143
ENSRNOT00000024102        AVFYVAAQLVGAIAGAGILYWLAPLNARGNLAVN---ALNNNTTPGKAMVVELILTFQLA 143
ENSMUST00000088200        AIFYVAAQLVGAIAGAGILYWLAPGNARGNLAVN---AQQHNTRQGRGGGVNLDFPAGPL 143
ENSRNOT00000040874        AVFYVAAQLVGAIAGAGILYWLAPLNARGNLAVNAVTEQQHNAWQGHGGGVNLDFPASPL 146
ENST00000293599           AFFYVAAQLVGAIAGAGILYGVAPLNARGNLAVN---ALNNNTTQGQAMVVELILTFQLA 143
ENSGALT00000041244        TLFYVIAQLVGAIAGAGILYGVTPANTRGNLAIN---SLNNNTTPGQAVVVEMILTFQLA 124
ENSGALT00000041246        TLFYVIAQLVGAIAGAGILYGVTPANTRGNLAIN---SLNNNTTPGQAVVVEMILTFQLA 140
ENSGALT00000004288        TLFYVIAQLVGAIAGAGILYGVTPANTRGNLAIN---SLNNNTTPGQAVVVEMILTFQLA 177
                          :.*** ************** ::* *:*****:*     .:*:  *:.  *:: :.

ENSMUST00000042340        LCIFSSTDSRRTSPVGSPALSIGLSVTLGHLVGIYFTGCSMNPARSFGPAVVMNRFSPSH 203
ENSRNOT00000024102        LCIFSSTDSRRTSPVGSPALSIGLSVTLGHLVGIYFTGCSMNPARSFGPAVVMNRFSPSH 203
ENSMUST00000088200        HLLLHGLPP--HQPGGLPSLIHWLVGHTGPSCGDLLHRLFHEPSPIFRPCGGHESVQPLS 201
ENSRNOT00000040874        HLLLHRLSP--NQPCGLPSLIHWLVCHTGPSCGDLLHRLFHEPSPIFRPCGGHEPVQPLS 204
ENST00000293599           LCIFASTDSRRTSPVGSPALSIGLSVTLGHLVGIYFTGCSMNPARSFGPAVVMNRFSPAH 203
ENSGALT00000041244        ACIFASTDNRRNGNVGSPALSIGLSVAVGHLVGVSVRGGTIGWKKSMEPSVTKKERSIAV 184
ENSGALT00000041246        ACIFASTDNRRNGNVGSPALSIGLSVAVGHLVGVSVRG---GWGRNFS-RMPILILSPIA 196
ENSGALT00000004288        ACIFASTDNRRNGNVGSPALSIGLSVAVGHLVGL--------YCCQLNPAKEVAQSSPPW 229
                            ::           * *:*   *    *   *             :         .

ENSMUST00000042340        WVFWVGPIVGAVLAAILYFYLLFPSSLSLHDRVAVVKGTYEPEEDWEDHREERKKTIELT 263
ENSRNOT00000024102        WVFWVGPIVGAMLAAILYFYLLFPSSLSLHDRVAVVKGTYEPEEDWEDHREERKKTIELT 263
ENSMUST00000088200        LGLLGRTIVGAVLAAILYFYLLFPSSLSLHDRVAVVKGTYEPEEDWEDHREERKKTIELT 261
ENSRNOT00000040874        LGLLGR-IVGAMLAAILYFYLLFPSSLSLHDRVAVVKGTYEPEEDWEDHREERKKTIELT 263
ENST00000293599           WVFWVGPIVGAVLAAILYFYLLFPNSLSLSERVAIIKGTYEPDEDWEEQREERKKTMELT 263
ENSGALT00000041244        CVFWVGPILGACLAALLYFYILVPYCMNMSDRVAIVKGTYESEEEWEEQREERKKSMELT 244
ENSGALT00000041246        FVFWVGPILGACLAALLYFYILVPYCMNMSDRVAIVKGTYESEEEWEEQREERKKSMELT 256
ENSGALT00000004288        WVFWVGPILGACLAALLYFYILVPYCMNMSDRVAIVKGTYESEEEWEEQREERKKSMELT 289
                            :    *:** ***:****:*.* .:.: :***::*****.:*:**::******::***

ENSMUST00000042340        AH 265
ENSRNOT00000024102        AH 265
ENSMUST00000088200        AH 263
ENSRNOT00000040874        AH 265
ENST00000293599           TR 265
ENSGALT00000041244        PP 246
ENSGALT00000041246        PP 258
ENSGALT00000004288        PP 291
                          .
```

AQP6 - CLUSTAL W (1.83) multiple sequence alignment

```
ENSRNOT00000000323      MEPGLCNRAYLLVGGLWTAISKALFAEFLATGLYVFFGVGSVLPWPVALPSVLQVAITFN 60
ENSRNOT00000049837      MEPGLCNRAYLLVGGLWTAISKALFAEFLATGLYVFFGVGSVLPWPVALPSVLQVAITFN 60
ENSMUST00000023754      MEPGLCSRAYLLVGGLWTAISKALFAEFLATGLYVFFGVGSVLPWPVALPSVLQIAITFN 60
ENSGALT00000016693      ------------------------------------------------------------
ENST00000315520         ------------------------------------------------------------
ENST00000394984         ------------------------------------------------------------
ENST00000394985         ------------------------------------------------------------


ENSRNOT00000000323      LATATAVQISWKTSGAHANPAVTLAYLVGSHISLPRAVAYIAAQLAGATVGAALLYGVTP 120
ENSRNOT00000049837      LATATAVQISWKTSGAHANPAVTLAYLVGSHISLPRAVAYIAAQLAGATVGAALLYGVTP 120
ENSMUST00000023754      LATATAVQISWKTSGAHANPAVTLAYLVGSHISLPRAMAYIAAQLAGATAGAALLYGVTP 120
ENSGALT00000016693      ------------------------------------------------------------
ENST00000315520         -----------------------------------------------------------M 1
ENST00000394984         -----------------------------------------------------------M 1
ENST00000394985         -----------------------------------------------------------M 1


ENSRNOT00000000323      GGVRETLGVNVVHNSTSTGQAVAVELVLTLQLV-LCVFASMDSRQ--TLGSP-----AAM 172
ENSRNOT00000049837      GGVRETLGVNVVHNSTSTGQAVAVELVLTLQLV-LCVFASMDSRQ--TLGSP-----AAM 172
ENSMUST00000023754      GGIRETLGVNVVHNSTSTGQAVAVELVLTLQLV-LCVFASMDGRQ--TLASP-----AAM 172
ENSGALT00000016693      -----------LHNETTTGQAVTVELFLTFQLV-LCIFASTDERREDNMGSP-----ALS 43
ENST00000315520         DAVEPGGRGWASMLACRLWKAISRALFAEFLATGLYVFFGVGSVMRWPTALPSVLQIAIT 61
ENST00000394984         DAVEPGGRGWASMLACRLWKAISRALFAEFLATGLYVFFGVGSVMRWPTALPSVLQIAIT 61
ENST00000394985         DAVEPGGRGWASMLACRLWKAISRALFAEFLATGLYVFFGVGSVMRWPTALPSVLQIAIT 61
                                           :*::  *.  :  . * :* . .       . *     *

ENSRNOT00000000323      IGTSVALGHLIGIYFTGCSMNPARSFGPAVIVG---KFAVHWIFWVGPLTGAVL-ASLIY 228
ENSRNOT00000049837      IGTSVALGHLIGIYFTGCSMNPARSFGPAVIVG---KFAVHWIFWVGPLTGAVL-ASLIY 228
ENSMUST00000023754      IGTSVALGHLIGIYFTGCSMNPARSFGPAVIVG---KFAVHWIFWVGPLTGAVL-ASLIY 228
ENSGALT00000016693      IGLSVAVGHLLGIRYTGCSMNPARSFAPAVIVG---DFSDHWVFWVGPLVGAAA-ASIIY 99
ENST00000315520         FNLVTAMAVQVTWKASGAHANPAVTL--AFLVGSHISLPRAVAYVAAQLVGATVGAALLY 119
ENST00000394984         FNLVTAMAVQVTWKASGAHANPAVTL--AFLVGSHISLPRAVAYVAAQLVGATVGAALLY 119
ENST00000394985         FNLVTAMAVQVTWKASGAHANPAVTL--AFLVGSHISLPRAVAYVAAQLVGATVGAALLY 119
                        :.  .*:.  :    :*.  *** ::  *.:**   .:.    : .. *.**.  *:::*

ENSRNOT00000000323      NFILFPDTKTVAQRLAILVGTT----------KVEKVVDLEPQKKE-------------- 264
ENSRNOT00000049837      NFILFPDTKTVAQRLAILVGTT----------KVEKVVDLEPQKKE-------------- 264
ENSMUST00000023754      NFILFPDTKTVAQRLAILVGTT----------KVEKVVDLEPQKKE-------------- 264
ENSGALT00000016693      NYVLFPQSKTFSERLAIFKG-------------------MEPEEDW-------------- 126
ENST00000315520         GVMPGDIRETLGINVVRNSVSTGQAVAVELLLTLQLVLCVFASTDSRQTSGSPATMIGIS 179
ENST00000394984         GVMPGDIRETLGINVAPPTSS---------------ALRLFPSSS--------------- 149
ENST00000394985         GVMPGDIRETLGINVVRNSVSTGQAVAVELLLTLQLVLCVFASTDSRQTSGSPATMIGIS 179
                        . :     :*.. .:.                       : .. .

ENSRNOT00000000323      --------SQTNSEDTEVSV---------------------------------------- 276
ENSRNOT00000049837      --------SQTNSEDTEVSV---------------------------------------- 276
ENSMUST00000023754      --------SQTNSEDTECLTSPCEEAVRSFSFTLGLC----------------------- 293
ENSGALT00000016693      --------AEREA----------------------------------------------- 131
ENST00000315520         VALGHLIGIHFTGCSMNPARSFGPAIIIGKFTVHWVFWVGPLMGALLASLIYNFVLFPDT 239
ENST00000394984         --------LHLEACLTRP------------FSIS-------------------------- 163
ENST00000394985         VALGHLIGIHFTGCSMNPARSFGPAIIIGKFTVHWVFWVGPLMGALLASLIYNFVLFPDT 239
                                 .  .

ENSRNOT00000000323      -------------------------------------------
ENSRNOT00000049837      -------------------------------------------
ENSMUST00000023754      -------------------------------------------
ENSGALT00000016693      -------------------------------------------
ENST00000315520         KTLAQRLAILTGTVEVGTGAGAGAEPLKKESQPGSGAVEMESV 282
ENST00000394984         -------------------------------------------
ENST00000394985         KTLAQRLAILTGTVEVGTGAGAGAEPLKKESQPGSGAVEMESV 282
```

AQP7 - CLUSTAL W (1.83) multiple sequence alignment

```
ENSMUST00000030136        ----------------MAPRSVLETIQSVL--QKNMVREFLAEFLSTYVMMVFGLGSVAH 42
ENSMUST00000054945        ----------------MAPRSVLETIQSVL--QKNMVREFLAEFLSTYVMMVFGLGSVAH 42
ENSMUST00000116345        ----------------MAPRSVLETIQSVL--QKNMVREFLAEFLSTYVMMVFGLGSVAH 42
ENSRNOT00000012975        ----------------MAG-SVLENIQSVL--QKTWVREFLAEFLSTYVLMVFGLGSVAH 41
ENST00000297988           MVQASGHR-RSTRGSKMVSWSVIAKIQEIL--QRKMVREFLAEFMSTYVMMVFGLGSVAH 57
ENST00000379507           --MGSGHCLRSTRGSKMVSWSVIAKIQEIL--QRKMVREFLAEFMSTYVMMVFGLGSVAH 56
ENST00000379506           --MGSGHCLRSTRGSKMVSWSVIAKIQEIL--QRKMVREFLAEFMSTYVMMVFGLGSVAH 56
ENST00000379503           -----------------MGWATFTD-------QGEEGLEPSGPWKTSHTR---------- 26
ENSGALT00000003866        ---------------------MLEKIQKMLTIRSNSVRELLAEALGMYILMVLGLSSVAQ 39
                                                :         :     *  .     :

ENSMUST00000030136        MVLGE-NSGSYLGVNLGFGFGVTMGVHVAGGISGAHMNAAVTFTNCALGRMTWKKFPVYV 101
ENSMUST00000054945        MVLGE-NSGSYLGVNLGFGFGVTMGVHVAGGISGAHMNAAVTFTNCALGRMTWKKFPVYV 101
ENSMUST00000116345        MVLGE-NSGSYLGVNLGFGFGVTMGVHVAGGISGAHMNAAVTFTNCALGRMTWKKFPVYV 101
ENSRNOT00000012975        MVLGE-RLGSYLGVNLGFGFGVTMGIHVAGGISGAHMNAAVTFTNCALGRMAWKKFPIYV 100
ENST00000297988           MVLNK-KYGSYLGVNLGFGFGVTMGVHVAGRISGAHMNAAVTFANCALGRVPWRKFPVYV 116
ENST00000379507           MVLNK-KYGSYLGVNLGFGFGVTMGVHVAGRISGAHMNAAVTFANCALGRVPWRKFPVYV 115
ENST00000379506           MVLNK-KYGSYLGVNLGFGFGVTMGVHVAGRISGAHMNAAVTFANCALGRVPWRKFPVYV 115
ENST00000379503           ----------------------------------AHMNAAVTFANCALGRVPWRKFPVYV 52
ENSGALT00000003866        TVLGKGEFGQYLSINLGFGIGVTMGIHAAGGISGAHLNAAITLTHCMFGNLPWRKLPVYL 99
                                                            **:***:*:::* :*.:.*:*:*:*:

ENSMUST00000030136        LGQFLGSFSAAATTYLIFYGAINHFAGGDLLVTGSKATANIFATYLPEYMTLWRGFLDEA 161
ENSMUST00000054945        LGQFLGSFSAAATTYLIFYGAINHFAGGDLLVTGSKATANIFATYLPEYMTLWRGFLDEA 161
ENSMUST00000116345        LGQFLGSFSAAATTYLIFYGAINHFAGGDLLVTGSKATANIFATYLPEYMTLWRGFLDEA 161
ENSRNOT00000012975        LGQFLGSFLAAATTYLIFYGAINHYAGGELLVTGPKSTANIFATYLPEHMTLWRGFVDEV 160
ENST00000297988           LGQFLGSFLAAATIYSLFYTAILHFSGGQLMVTGPVATAGIFATYLPDHMTLWRGFLNEA 176
ENST00000379507           LGQFLGSFLAAATIYSLFYTAILHFSGGQLMVTGPVATAGIFATYLPDHMTLWRGFLNEA 175
ENST00000379506           LGQFLGSFLAAATIYSLFYTAILHFSGGQLMVTGPVATAGIFATYLPDHMTLWRGFLNEA 175
ENST00000379503           LGQFLGSFLAAATIYSLFYTAILHFSGGQLMVTGPVATAGIFATYLPDHMTLWRGFLNEA 112
ENSGALT00000003866        LGQFLGSFLAAATIFGLYYDALHDYTNGNFTVTGPTATASIFSTYPSPYMSLTGGFFTEF 159
                          ******** **** : ::* *: .::.*:: ***. :**.**:** . :*:*  **. *

ENSMUST00000030136        FVTGMLQLCLFAITDKKNSPALQGTEPLVIGILVTVLGVSLGMNSGYAINPSRDLPPRLF 221
ENSMUST00000054945        FVTGMLQLCLFAITDKKNSPALQGTEPLVIGILVTVLGVSLGMNSGYAINPSRDLPPRLF 221
ENSMUST00000116345        FVTGMLQLCLFAITDKKNSPALQGTEPLVIGILVTVLGVSLGMNSGYAINPSRDLPPRLF 221
ENSRNOT00000012975        FVTGMLQLCIFAITDKLNSPALQGTEPLMIGILVCVLGVSLGMNTGYAINPSRDLPPRFF 220
ENST00000297988           WLTGMLQLCLFAITDQENNPALPGTEALVIGILVVIIGVSLGMNTGYAINPSRDLPPRIF 236
ENST00000379507           WLTGMLQLCLFAITDQENNPALPGTEALVIGILVVIIGVSLGMNTGYAINPSRDLPPRIF 235
ENST00000379506           WLTGMLQLCLFAITDQENNPALPGTEALVIGILVVIIGVSLGMNTGYAINPSRDLPPRIF 235
ENST00000379503           WLTGMLQLCLFAITDQENNPALPGTEALVIGILVVIIGVSLGMNTGYAINPSRDLPPRIF 172
ENSGALT00000003866        IATVMLLLGILVIHDEKNNGALKGTHALLTGILVLGIGMGMGMNTGYAINPSRDLPPRIF 219
                            * ** * ::.* *: *. ** **..*: ****  :*:.:***:*************:*

ENSMUST00000030136        TFIAGWGKQVFRAGNNWWWVPVVAPLLGAYLGGIVYLGLIHPSIPQDPQRLENFTARDQK 281
ENSMUST00000054945        TFIAGWGKQVFRAGNNWWWVPVVAPLLGAYLGGIVYLGLIHPSIPQDPQRLENFTARDQK 281
ENSMUST00000116345        TFIAGWGKQVFRAGNNWWWVPVVAPLLGAYLGGIVYLGLIHPSIPQDPQRLENFTARDQK 281
ENSRNOT00000012975        TFIAGWGKKVFSAGNNWWWVPVVAPLLGAYLGGIVYLGLIHAGIP--PQ----------- 267
ENST00000297988           TFIAGWGKQVFSNGENWWWVPVVAPLLGAYLGGIIYLVFIGSTIPREPLKLEDSVAYEDH 296
ENST00000379507           TFIAGWGKQVFSNGENWWWVPVVAPLLGAYLGGIIYLVFIGSTIPREPLKLEDSVAYEDH 295
ENST00000379506           TFIAGWGKQVFR------YCPCPGPFL--------------------------------- 256
ENST00000379503           TFIAGWGKQVFR------YCPCPGPFL--------------------------------- 193
ENSGALT00000003866        TAIAGWGVDVFRAGNYWWWVPVVAPTLGSLAGGLIHKLLID--IHNQPVLERGNEKGQTV 277
                          * ***** .**       : *  .* *

ENSMUST00000030136        VTASYKNAASANISGSVPLEHF------------------------ 303
ENSMUST00000054945        VTASYKNAASANISGSVPLEHF------------------------ 303
ENSMUST00000116345        VTASYKNAASANISGSVPLEHF------------------------ 303
ENSRNOT00000012975        --------------GS------------------------------ 269
ENST00000297988           GITVLPKMGSHEPTISPLTPVSVSPANRSSVHPAPPLHESMALEHF 342
ENST00000379507           GITVLPKMGSHEPTISPLTPVSVSPANRSSVHPAPPLHESMALEHF 341
ENST00000379506           ----------------------------------------------
ENST00000379503           ----------------------------------------------
ENSGALT00000003866        VESS------------------------------------------ 281
```

AQP8 - CLUSTAL W (1.83) multiple sequence alignment

```
ENSMUST00000033023      MSGEQTPMCSMDLPEVKVKTS--MAGRCRVFWYEQYVQPCIVELVGSALFIFIGCLSVIE 58
ENSMUST00000098056      MPAEHAPTS--------------------CFCSQLYR----------------------- 17
ENSRNOT00000019939      FSLRQTPMCSMDLREIKGKETNMADSYHGMSWYEQYIQPCVVELLGSALFIFIGCLSVIE 60
ENST00000219660         MSG-EIAMCEPEFGNDKAREPS-VGGRWRVSWYERFVQPCLVELLGSALFIFIGCLSVIE 58
ENSGALT00000009629      LGGQPSTKEAMEMVDAERPPAK-------QNWYECYVLPCLAELLGSAAFVFIGCLSVVE 53
                        :     .                          : :

ENSMUST00000033023      NSPNTGLLQPALAHGLALGLIIATLGNISGGHFNPAVSLAVTVIGGLKTMLLIPYWISQL 118
ENSMUST00000098056      ------------------------LG----GHFNPAVSLAVTVIGGLKTMLLIPYWISQL 49
ENSRNOT00000019939      NSPNTGLLQPALAHGLALGLIIATLGNISGGHFNPAVSLAVTLVGGLKTMLLIPYWVSQL 120
ENST00000219660         NGTDTGLLQPALAHGLALGLVIATLGNISGGHFNPAVSLAAMLIGGLNLVMLLPYWVSQL 118
ENSGALT00000009629      DSIGTGLLQPALVHGLSIVPIVVSLGNISGAHINPVVSLGIWLVGGMKLIMLIPYCIAQL 113
                                                **    .*:**.***.  ::**:: ::*:** ::**

ENSMUST00000033023      FGGLIGAALAKVVSPEERFWNASGAAFAIVQEQEQVAEALGIEIILTMLLVLAVCMGAVN 178
ENSMUST00000098056      FGGLIGAALAKVVSPEERFWNASGAAFAIVQEQEQVAEALGIEIILTMLLVLAVCMGAVN 109
ENSRNOT00000019939      FGGMIGAALAKVVSPEERFWNASGAAFAIVQEQEQVAEALGVEIVMTMLLVLAVCMGAVN 180
ENST00000219660         LGGMLGAALAKAVSPEERFWNASGAAFVTVQEQGQVAGALVAEIILTTLLALAVCMGAIN 178
ENSGALT00000009629      CGGILGAALTKAVASSLNFDSRVGGAFGIIRTNAQIGPALGNEIILTTFLLLVVCMTAVN 173
                         **::****:*.*:.. .* .  *.**  :: : *:. **  **::* :* *.*** *:*

ENSMUST00000033023      EKTMGPLAPFSIGFSVIVDILAGGSISGACMNPARAFGPAVMAGYWDFHWIYWLGPLLAG 238
ENSMUST00000098056      EKTMGPLAPFSIGFSVIVDILAGGSISGACMNPARAFGPAVMAGYWDFHWIYWLGPLLAG 169
ENSRNOT00000019939      EKTMGPLAPFSIGFSVIVDILAGGGISGACMNPARAFGPAVMAGYWDFHWIYWLGPLLAG 240
ENST00000219660         EKTKGPLAPFSIGFAVTVDILAGGPVSGGCMNPARAFGPAVVANHWNFHWIYWLGPLLAG 238
ENSGALT00000009629      GETKSPLAPVCIALTVVINIIVGGSISGPCMNPARAFGPAVIANYWLYHWVYWVGPLIGC 233
                         :* .****..*.::* ::*:.** :** ************:*.:* :**:**:***:.

ENSMUST00000033023      LFVGLLIRLLIGDEKTRLILKSR 261
ENSMUST00000098056      LFVGLLIRLLIGDEKTRLILKSR 192
ENSRNOT00000019939      LFVGLLIRLFIGDEKTRLILKSR 263
ENST00000219660         LLVGLLIRCFIGDGKTRLILKAR 261
ENSGALT00000009629      LISSLLIRFVIGDRKIRLFLK-- 254
                        *: .**** .*** * **:**
```

AQP9 - CLUSTAL W (1.83) multiple sequence alignment

```
ENSMUST00000113569      --------------------MPSEKDRAKKNLVQRLALKSCLAKETLSEFLGTF------ 34
ENSMUST00000113570      --------------------MPSEKDRAKKNLVQRLALKSCLAKETLSEFLGTF------ 34
ENSMUST00000074465      --------------------MPSEKDRAKKNLVQRLALKSCLAKETLSEFLGTF------ 34
ENSMUST00000060917      MAWQFYLFVCLFVYLFIYVCAPMHTSASENGLLESALLPPSFHHVDSRDGAQVIRPGNRD 60
ENSRNOT00000021442      --------------------MPSEKDGAKKSLMQRLALKSRIAKETLSEFLGTF------ 34
ENST00000219919         --------------------MQPEGAEKGKSFKQRLVLKSSLAKETLSEFLGTF------ 34
ENSGALT00000006776      ------------------------------------------------------------
ENSGALT00000039255      -------------------EEAKMSRKNKRSLKEKFALKNSLVKEALSEFLGTF------ 35


ENSMUST00000113569      IMIVLGCGSIAQAVLSREKAGGIITINIGFATAVVMA-LYATFGVSGGHINPAVSFAMCT 93
ENSMUST00000113570      IMIVLGCGSIAQAVLSREKAGGIITINIGFATAVVMA-LYATFGVSGGHINPAVSFAMCT 93
ENSMUST00000074465      IMIVLGCGSIAQAVLSREKAGGIITINIGFATAVVMA-LYATFGVSGGHINPAVSFAMCT 93
ENSMUST00000060917      FYLVLGCGSIAQAVLSREKAGGIITINIGFATAVVMA-LYATFGVSGGHINPAVSFAMCT 119
ENSRNOT00000021442      IMIVLGCSSIAQAVLSRERFGGIITINIGFASAVVMA-LYVTFGISGGHINPAVSFAMCA 93
ENST00000219919         ILIVLGCGCVAQAILSRGRFGGVITINVGFSMAVAMA-IYVAGGVSGGHINPAVSLAMCL 93
ENSGALT00000006776      ----------------------------GSSIAVYTCGVYVGGNFSGGHINPAVSLAMCV 32
ENSGALT00000039255      IMITLGCGCVGQAVLSRGAHGGPMTISVGFAMAVTTA-VYVSGGVSGGHINPAVSLAMCV 94
                                                    * : **  . :*.  ..**********:***

ENSMUST00000113569      FGRMEWFKFPFYVGAQLLGAFVGAATVFGIYYDGLMAFADGKLLITGENGTAFIFATYPK 153
ENSMUST00000113570      FGRMEWFKFPFYVGAQLLGAFVGAATVFGIYYDGLMAFADGKLLITGENGTAFIFATYPK 153
ENSMUST00000074465      FGRMEWFKFPFYVGAQLLGAFVGAATVFGIYYDGLMAFADGKLLITGENGTAFIFATYPK 153
ENSMUST00000060917      FGRMEWFKFPFYVGAQLLGAFVGAATVFGIYYDGLMAFADGKLLITGENGTAFIFATYPK 179
ENSRNOT00000021442      FGRMEWFKFPFYVGAQFLGAFVGAATVFGIYYDGLMAFAGGKLLVVGENATAFIFATYPA 153
ENST00000219919         FGRMKWFKLPFYVGAQFLGAFVGAATVFGIYYDGLMSFAGGKLLIVGENATAHIFATYPA 153
ENSGALT00000006776      AGRLKWTKLPIYILAQFLGAFVGAAAVFGIYHDAFMEYSNGTLEVTGPYATAHIFATYPA 92
ENSGALT00000039255      AGRLKWTKLPIYILAQFLGAFVGAAAVFGIYHDAFMEYSNGTLEVTGPYATAHIFATYPA 154
                         **::* *:*:*: **:********:*****:*.:* ::.*.* :.*  .**.******

ENSMUST00000113569      PFVSVPGAFVDQSP---------------------------------------------- 167
ENSMUST00000113570      PFVSVPGAFVDQVVSTMFLLLIVFAIFDSRNLGVPRGLEPIVIGLLIIVISCSLGLNSGC 213
ENSMUST00000074465      PFVSVPGAFVDQVVSTMFLLLIVFAIFDSRNLGVPRGLEPIVIGLLIIVISCSLGLNSGC 213
ENSMUST00000060917      PFVSVPGAFVDQVVSTMFLLLIVFAIFDSRNLGVPRGLEPIVIGLLIIVISCSLGLNSGC 239
ENSRNOT00000021442      PFISTPGAFVDQVVSTMFLLLIVFAMFDSRNLGVPRGLEPVVIGLLIIVLSCSLGLNSGC 213
ENST00000219919         PYLSLANAFADQVVATMILLIIVFAIFDSRNLGAPRGLEPIAIGLLIIVIASSLGLNSGC 213
ENSGALT00000006776      PYLSLINGFADQVMSTAVLLLVIFAIFDTRNNRVPKGLEPVVVGLLIIVLTCSLGMNSGC 152
ENSGALT00000039255      PYLSLINGFADQVMSTAVLLLVIFAIFDTRNNRVPKGLEPVVVGLLIIVLTCSLGMNSGC 214
                        *::*  ..*.**

ENSMUST00000113569      --------WPRRITHIKGS-----------------SRILSAIRG--------------- 187
ENSMUST00000113570      AMNPARDLSPRLFTALAGWGFEVFTFGNNFWWIPVVGPMIGAVLGGLIYVLFIQMHHSNP 273
ENSMUST00000074465      AMNPARDLSPRLFTALAGWGFEVFTFGNNFWWIPVVGPMIGAVLGGLIYVLFIQMHHSNP 273
ENSMUST00000060917      AMNPARDLSPRLFTALAGWGFEVFTFGNNFWWIPVVGPMIGAVLGGLIYVLFIQMHHSNP 299
ENSRNOT00000021442      AMNPARDLSPRLFTALAGWGFEVFTVGNNFWWIPVVGPMIGAFLGGLIYILFIQMHHSKL 273
ENST00000219919         AMNPARDLSPRLFTALAGWGFEVFRAGNNFWWIPVVGPLVGAVIGGLIYVLVIEIHHPEP 273
ENSGALT00000006776      AMNPARDLGPRLFTAIAGWGMEVFTAGNNWWWVPIVAPMLGGVLGALTYIIFIEVHHSD- 211
ENSGALT00000039255      AMNPARDLGPRLFTAIAGWGMEVFTAGNNWWWVPIVAPMLGGVLGALTYIIFIEVHHSD- 273
                                 ** :* : *                  . ::... *

ENSMUST00000113569      ---VEHRP-------------- 192
ENSMUST00000113570      DPEVKAEPAENNLEKHELSVIM 295
ENSMUST00000074465      DPEVKAEPAENNLEKHELSVIM 295
ENSMUST00000060917      DPEVKAEPAENNLEKHELSVIM 321
ENSRNOT00000021442      DPDMKAEPSENNLEKHELSVIM 295
ENST00000219919         DSVFKTEQSEDKPEKYELSVIM 295
ENSGALT00000006776      ---NQQGEENDVYDKYELTNM- 229
ENSGALT00000039255      ---NQQGEENDVYDKYELTNM- 291
                            :
```

AQP10 - CLUSTAL W (1.83) multiple sequence alignment

```
ENST00000324978      MVFTQAPAEIMGHLRIRSLLARQCLAEFLGVFVLMLLTQGAVAQAVTSGETKGNFFTMFL 60
ENST00000355197      MVFTQAPAEIMGHLRIRSLLARQCLAEFLGVFVLMLLTQGAVAQAVTSGETKGNFFTMFL 60
                     ************************************************************

ENST00000324978      AGSLAVTIAIYVGGNVSGAHLNPAFSLAMCIVGRLPWVKLPIYILVQLLSAFCASGATYV 120
ENST00000355197      AGSLAVTIAIYVGGNVSGAHLNPAFSLAMCIVGRLPWVKLPIYILVQLLSAFCASGATYV 120
                     ************************************************************

ENST00000324978      LYHDALQNYTGGNLTVTGPKETASIFATYPAPYLSLNNGFLDQVLGTGMLIVGLLAILDR 180
ENST00000355197      LYHDALQNYTGGNLTVTGPKETASIFATYPAPYLSLNNGFLDQVLGTGMLIVGLLAILDR 180
                     ************************************************************

ENST00000324978      RNKGVPAGLEPVVVGMLILALGLSMGANCGIPLNPARDLGPRLFTYVAGWGPEVFSAGNG 240
ENST00000355197      RNKGVPAGLEPVVVGMLILALGLSMGANCGIPLNPARDLGPRLFTYVAGWGPEVFRWET- 239
                     *******************************************************   .

ENST00000324978      WWWVPVVAPLVGATVGTATYQLLVALHHPEGPEPAQDLVSAQHKASELETPASAQMLECK 300
ENST00000355197      ------------DSPGAG-------LHSPS---------SAKG-----SVPGSTALCL-- 264
                                  : *:.       ** *.         **:      ..*.*: :

ENST00000324978      L 301
ENST00000355197      -
```

AQP11 - CLUSTAL W (1.83) multiple sequence alignment

```
ENSRNOT00000018091      MSALLGLPPEVQDTCISLGLMLLVVLFMGLARVIARQQLH-RPMVHAFVLEFLATFQLCY 59
ENSRNOT00000038705      MSALLGLPPEVQDTCISLGLMLLVVLFMGLARVIARQQLH-RPMVHAFVLEFLATFQLCY 59
ENSMUST00000055379      MSALLGLRPEVQDTCISLGLMLLFVLFVGLARVIARQQLH-RPVVHAFVLEFLATFQLCC 59
ENSMUST00000084986      MSALLGLRPEVQDTCISLGLMLLFVLFVGLARVIARQQLH-RPVVHAFVLEFLATFQLCC 59
ENST00000313578         MSPLLGLRSELQDTCTSLGLMLSVVLLMGLARVVARQQLH-RPVAHAFVLEFLATFQLCC 59
ENSGALT00000002458      ASGTVAMAAG--GIWMSLGLMAAVVAAVGLCRSLALRRLHARPHLCSFLLELLSTFQVCA 58
                         *  :.: .   .   *****  .*  :**.* :* ::** **   :*:**:*:***:*

ENSRNOT00000018091      CTHELQLLSEQDSGHPTWTLTLIYFFSLVHGLTLVGTASNPCGVMMQMILGGMSPEMGAV 119
ENSRNOT00000038705      CTHELQLLSEQDSGHPTWTLTLIYFFSLVHGLTLVGTASNPCGVMMQMILGGMSPEMGAV 119
ENSMUST00000055379      CTHELQVLSEQDSAHPTWTLTLIYFFSLVHGLTLVGTASNPCGVMMQMILGGMSPEMGAV 119
ENSMUST00000084986      CTHELQVLSEQDSAHPTWTLTLIYFFSLVHGLTLVGTASNPCGVMMQMILGGMSPEMGAV 119
ENST00000313578         CTHELQLLSEQHPAHPTWTLTLVYFFSLVHGLTLVGTSSNPCGVMMQMMLGGMSPETGAV 119
ENSGALT00000002458      CTNELSLLGNTEP-RPHTALTLTYGFTVLHGLTLPGSTCNPCGTLQPLWGGRTSARAGGL 117
                        **:**.:*.: .. :*  :*** * *:::***** *::.****.:  :  *  *.. *.:

ENSRNOT00000018091      RLMAQLVSALCSRYCISALWSLSLTKYHFDERILACRNPINTDISKAIIIEAICSFIFHS 179
ENSRNOT00000038705      RLMAQLVSALCSRYCISALWSLSLTKYHFDERILACRNPINTDISKAIIIEAICSFIFHS 179
ENSMUST00000055379      RLLAQLVSALCSRYCISALWSLSLTKYHYDERILACRNPIHTDMSKAIIIEAICSFIFHS 179
ENSMUST00000084986      RLLAQLVSALCSRYCISALWSLSLTKYHYDERILACRNPIHTDMSKAIIIEAICSFIFHS 179
ENST00000313578         RLLAQLVSALCSRYCTSALWSLGLTQYHVSERSFACKNPIRVDLLKAVITEAVCSFLFHS 179
ENSGALT00000002458      KIWAQFAAAVLARVFMHFIWRLEMAESHLGALTQGCGNPMQTTEVQAFCIELLFSVVFQL 177
                        :: **:.:*: :*     :* * ::: * .    .* **:..   :*.  * : *.:*:

ENSRNOT00000018091      ALLHFQEVRTKLRIHVLAALITFLAYAGGSLTGALFNPALALSLHFPCFDESFYKFFVVY 239
ENSRNOT00000038705      ALLHFQEVRTKLRIHVLAALITFLAYAG-------------------------------- 207
ENSMUST00000055379      ALLHFQEVRTKLRIHLLAALITFLAYAG-------------------------------- 207
ENSMUST00000084986      ALLHFQEVRTKLRIHLLAALITFLAYAGGSLTGALFNPALALSLHFPCFDELFYKFFVVY 239
ENST00000313578         ALLHFQEVRTKLRIHLLAALITFLVYAGGSLTGAVFNPALALSLHFMCFDEAFPQFFIVY 239
ENSGALT00000002458      TILQVENIKPSYRVHAVALLITMLVFAG-------------------------------- 205
                        ::*:.::::.. *:* :* ***:*.:**

ENSRNOT00000018091      WVAPSLGVLLMILMFSFFLPWLHNNQLSNKKE 271
ENSRNOT00000038705      -------VLLMILMFSFFLPWLHNN------- 225
ENSMUST00000055379      -------VLMMILMFSFFLPWLHNNQMTNKKE 232
ENSMUST00000084986      WLAPSVGVLMMILMFSFFLPWLHNNQMTNKKE 271
ENST00000313578         WLAPSLGILLMILMFSFFLPWLHNNHTINKKE 271
ENSGALT00000002458      --------------------------------
```

AQP12 - CLUSTAL W (1.83) multiple sequence alignment

```
ENSMUST00000059676      MASLNVSLCFFFATCAICEVARRASKALLPAGTYASFARGAVGAAQLAACCLEMRVLVEL 60
ENSRNOT00000005935      MASLNVSLSFFFATCAICEVARRASKALLPAGTYASFAREAVGAAQLAACCLEMRVLVEL 60
ENSGALT00000010387      MAGLNVSIAFFFLVLGTCEAFRRLSKRLLAPGLLVCLTAELAGSLQLCSGCLELRMLLEI 60
ENST00000337801         MAGLNVSLSFFFATFALCEAARRASKALLPVGAYEVFAREA------------MRTLVEL 48
ENST00000373309         ---------------------NQPAPALSPQ----------------------------- 10
                                             .: :  * .

ENSMUST00000059676      GPWAGGFGPDLLLTLVFLLFLVHGVTFDGASANPTVALQEFLMVEASLPNTLLKLSAQVL 120
ENSRNOT00000005935      GPWAGGFGPDLLLTLVFLLFLVHGVTFDGASANPTVALQEFLMVEASLPSTLLKLLAQVL 120
ENSGALT00000010387      GPWGG-LSPDVVLTLLFLLFVVHAACANGAASNPTVSLQEFLLFECSLVATVAKLLAQCV 119
ENST00000337801         GPWAGDFGPDLLLTLLFLLFLAHGVTLDGASANPTVSLQEFLMAEQSLPGTLLKLAAQGL 108
ENST00000373309         ------------------------VSCRHSSSGGPGRWQEFLMAEQSLPGTLLKLAAQGL 46
                                                .    :::. .   ****: * **  *: ** ** :

ENSMUST00000059676      GAQAACALTQRCWAWELSELHLLQSLMAAHCSSTLRTSVLQGMLVEGACTFFFHLSLLHL 180
ENSRNOT00000005935      GAQAACALTQRCWTWELSELHLLQSLMAVHCSSTLRTSVLQGTLVEGACTFLFHLSLLHL 180
ENSGALT00000010387      GTGMGWALTKLYWSWELTQLHLIQNLMASECSSSIRTSPHLGALVEAACSFFFHLILLRV 179
ENST00000337801         GMQAACTLMRLCWAWELSDLHLLQSLMAQSCSSALRTSVPHGALVEAACAFCFHLTLLHL 168
ENST00000373309         GMQAACTLMRLCWAWELSDLHLLQSLMAQSCSSALRTSVPHGALVEAACAFCFHLTLLHL 106
                        *   . :* :  *:***::***:*.***  ***::***   * ***.**:* *** **::

ENSMUST00000059676      QHSLLVYRVPALALLVTLMAYTAGPYTSAFFNPALAASVTFHCPGNTLLEYAHVYCLGPV 240
ENSRNOT00000005935      QRSLLVYRAPALALLVTAMAYTAGPYTSAFFNPALAASVTFHCSGNTLLEYAHVYCLGPV 240
ENSGALT00000010387      RRSPAVCRVPVLAATVTFLTYVAAPATGAFFNPALATASTFLCAGSSLQDYIQVYWLGPL 239
ENST00000337801         RHSPPAYSGPAVALLVTVTAYTAGPFTSAFFNPALAASVTFACSGHTLLEYVQVYWLGPL 228
ENST00000373309         RHSPPAYSGPAVALLVTVTAYTAGPFTSAFFNPALAASVTFACSGHTLLEYVQVYWLGPL 166
                        ::*  .   *.:*  **  :*.*.* *.********:: ** *.* :* :* :** ***:

ENSMUST00000059676      AGMILAVLLHQGHLPR------LFQR-----NLFYRQKSKYRTPRGKLSPG--------- 280
ENSRNOT00000005935      AGMILAVLLHQGHLPR------FFER-----NLFYRQKSKYRTPRVKLSPG--------- 280
ENSGALT00000010387      AGMLAALLLYQGNIPR------FFQK-----NLLYGQKSKYKIPKARVMHA--------- 279
ENST00000337801         TGMVLAVLLHQGRLPH------LFQR-----NLFYGQKNKYRAPRGKPAPA--------- 268
ENST00000373309         TEEQHGEVMEPPLGPRSGDSGPWLQRPRWSPHLLLHLLSDTRLHLTEVPEGQLGSYGILE 226
                        :    . ::     *:       :::     :*:    .. :    .   .

ENSMUST00000059676      -------------SVDAKM-HKGE---------------- 290
ENSRNOT00000005935      -------------SVDTKM-LKGE---------------- 290
ENSGALT00000010387      -------------EDGEQQ-QKGK-----GEKSNPAPRA- 299
ENST00000337801         -------------SGDTQTPAKGSSVREPGRSGVEGPHSS 295
ENST00000373309         AFFSFCWLYTGEVSGAMPQPPLGGKDRSWGSLRHLALRW- 265
                                     .        *
```
