# Supplementary material for: Integrative sequence and tissue expression profiling of chicken and mammalian aquaporins
Source: BMC Genomics. 2009 Jul 14;10(Suppl 2):S7. doi: 10.1186/1471-2164-10-S2-S7 (PMC2966333; doi:10.1186/1471-2164-10-S2-S7)
Supplement: Additional file 2 — UniGene Transcripts per million and spot intensity of human, mouse and chicken AQP12 expressed in pancreas. [file 1471-2164-10-S2-S7-S2.docx]

| **Aquaporin Type** | **UniGene ID** | **Transcripts per Million (TPM)** | **Spot Intensity based on TPM** |
| --- | --- | --- | --- |
| AQP12 | Hs.437167 | 27 | 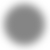 |
| AQP12 | Mm.235537 | 243 | 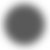 |
| AQP12 | Gga.19694 | 411 | 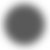 |
